# Supplementary material for: Exposure to the Russian Internet Research Agency foreign influence campaign on Twitter in the 2016 US election and its relationship to attitudes and voting behavior
Source: Nat Commun. 2023 Jan 9;14:62. doi: 10.1038/s41467-022-35576-9 (PMC9829855; doi:10.1038/s41467-022-35576-9)
Supplement: Supplementary file 1 — Supplementary Information [file 41467_2022_35576_MOESM1_ESM.pdf]

Exposure to the Russian Internet Research Agency foreign influence campaign on Twitter in the 2016 US Election and its relationship to attitudes and voting behavior

Supplementary Information

Contents

Supplementary Methods **2**
A Data . . . 2
B Equivalence testing for minimal relationships . . . 13
C Exposure to foreign influence accounts . . . 16
D Exposure to political actors . . . 23
E Vote change regression models . . . 24
F Linear models of ideological positioning . . . 39
G Adjustment for multiple hypothesis testing . . . 63

Supplementary References **65**

Supplementary Methods

A Data

A1 Twitter Elections Integrity releases

In the run-up to the 2018 US midterm elections, Twitter announced a new initiative designed to combat foreign-backed information campaigns.[1] As part of this initiative, the company continuously identified and blocked accounts of suspected foreign influence accounts. In addition to this, Twitter also released data that included the unique IDs of those accounts identified as foreign influence accounts and their country of origin.

To date, Twitter has released 27 Elections Integrity datasets that include the user profiles, tweets, and associated media from accounts that it classified as belonging to state-backed foreign influence campaigns. Overall, these datasets cover a wide range of national, political, and temporal contexts. Of those released to date, 17 of these datasets include at least one tweet from 2016 or earlier. In Supplementary Table A1, we present a summary of these releases covering the time period relevant to our research (2016 and earlier).

In Supplementary Figure A1, we also show the daily prevalence of tweets from different campaigns in the Elections Integrity data. Analogous to Panel A of Figure 1 in the main article, Supplementary Figure A1 demonstrates that the over-representation of tweets from accounts linked to Russia in our respondents' feeds is not a function of higher prevalence of this content in the datasets released by Twitter, but instead likely to result from a coordinated effort to target voters in the US prior to the 2016 election. Furthermore, Supplementary Figure A2 shows the potential daily outreach of foreign influence accounts' messages by aggregating the number of the original tweets as well as the number of times they were retweeted or quote-tweeted. As can be seen from the figure and table below, the Russian campaign had a noticeably higher virality than the rest of the foreign influence campaigns in that period. Some of the posted tweets received especially high traction, resulting in clear visible spikes in October and November of 2016.

The precise process by which Twitter identifies foreign influence accounts is not publicly detailed by Twitter. However, because Twitter has additional internal data about each account's behavior, and has an overall corporate interest in discovering these accounts, we assume that the company's identification is generally robust, unbiased, and of higher accuracy than could be done externally, especially considering that many these accounts are routinely suspended.

[1] https://blog.twitter.com/en_us/topics/company/2018/2016-election-update.html, https://blog.twitter.com/en_us/topics/company/2018/an-update-on-our-elections-integrity-work.html

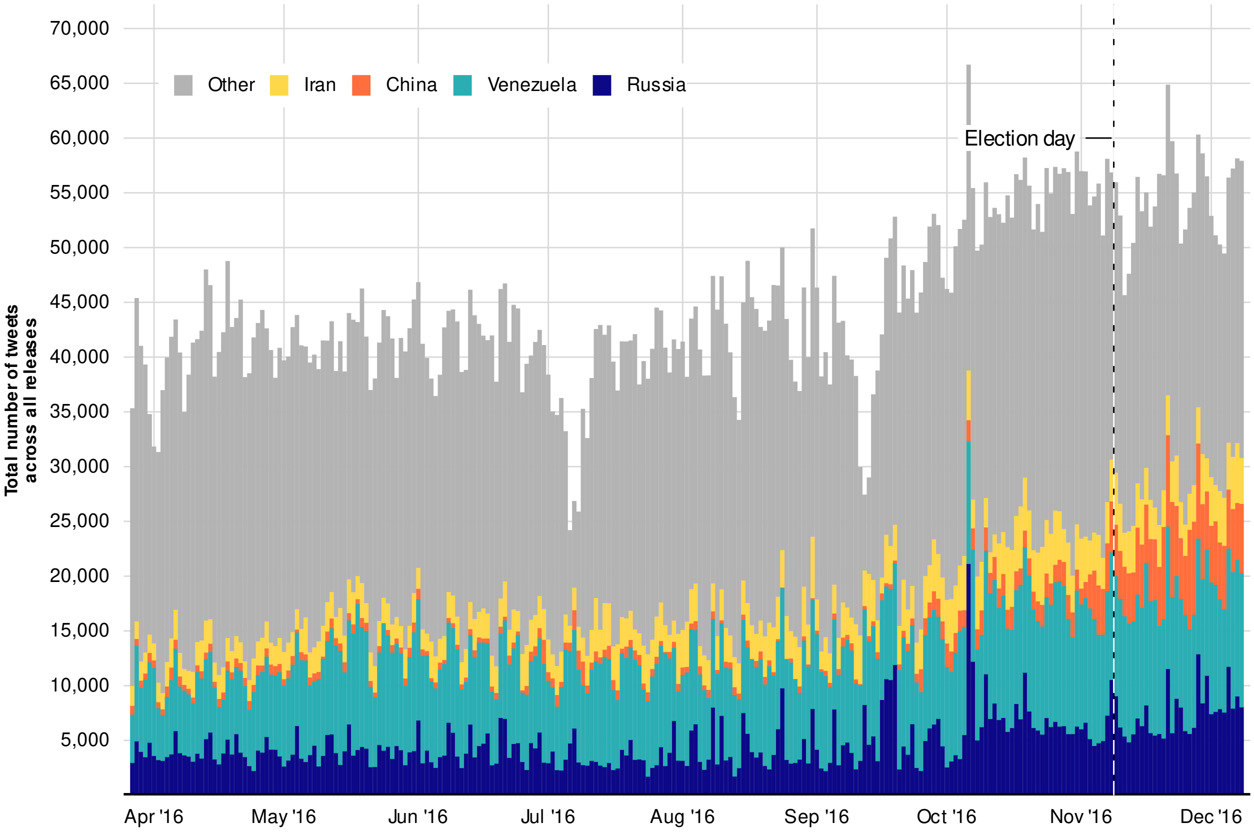

Supplementary Figure A1: Prevalence of tweets among surveyed respondents' Twitter feeds for each campaign in the Twitter Elections Integrity dataset (tweets only)

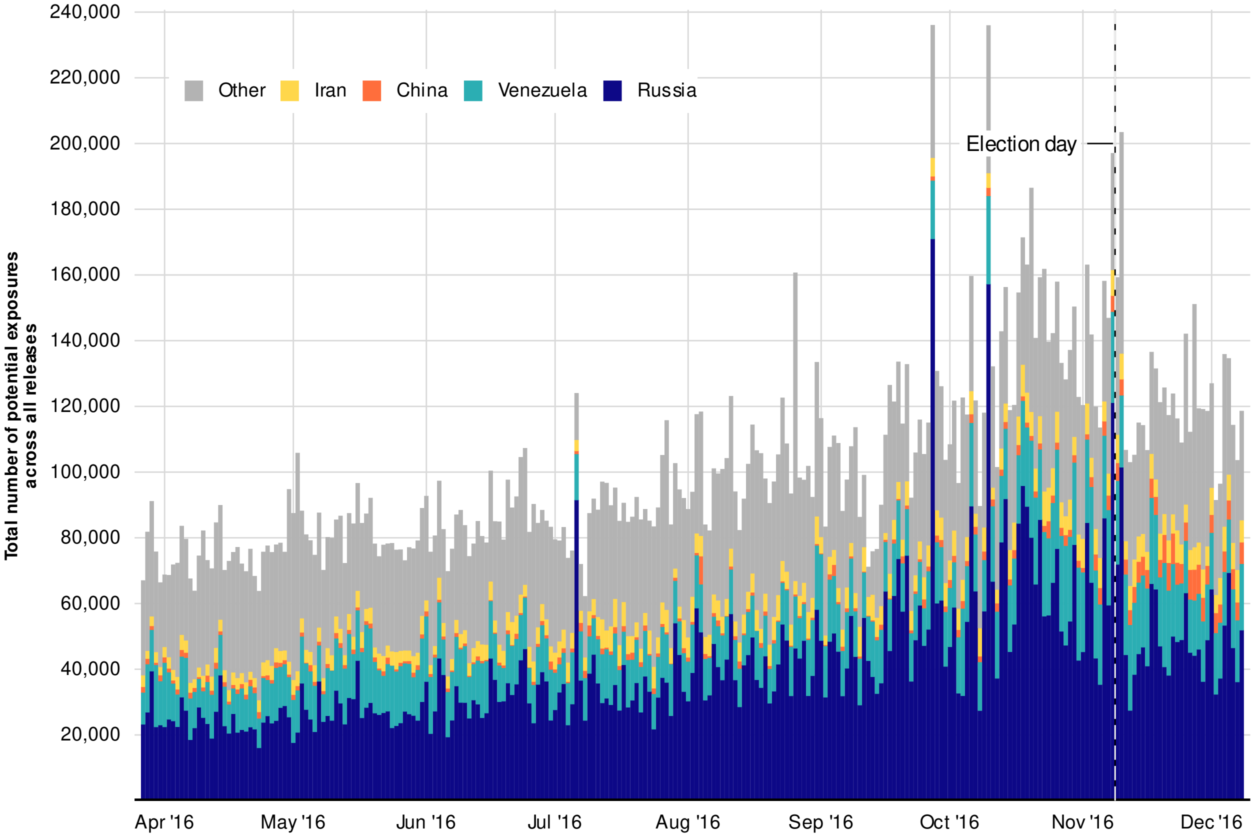

Supplementary Figure A2: Prevalence of tweets, retweets, and quote tweets among surveyed respondents' Twitter feeds for each campaign in the Twitter Elections Integrity dataset

Supplementary Table A1: Summary of the Twitter Elections Integrity Releases (covering 2016 and earlier)

						Retweets		Likes		Replies		Quotes	
Country Responsible	Data Released	N Tweets	N Users	Earliest Tweet	Last Tweet	Total	Mean	Total	Mean	Total	Mean	Total	Mean
Bangladesh	2019_01	6,852	4	2009-05-20	2016-12-31	475	0.0693228	1,660	0.2422650	137	0.0199942	2	0.0002919
Catalonia	2019_06	543	9	2011-11-24	2016-12-30	1,190	2.1915285	664	1.2228361	123	0.2265193	9	0.0165746
China	2019_08	2,247,774	300	2007-12-23	2016-12-31	1,057,857	0.4755957	453,391	0.2038374	231,220	0.1039528	4,574	0.0020564
Ecuador	2019_08	82,457	73	2010-03-13	2016-12-31	26,428	0.3205842	9,462	0.1147786	11,970	0.1452018	203	0.0024625
Egypt/SA/UAE	2019_08	70,207	92	2011-07-23	2016-12-31	80,002	1.1395647	78,078	1.1121589	12,318	0.1754601	2,072	0.0295140
Egypt/SA/UAE	2020_04	13,882,912	2,258	2008-04-17	2016-12-31	9,250,431	0.6669170	2,201,724	0.1587350	593,503	0.0427891	15,609	0.0011253
Ghana/Nigeria	2020_03	363	2	2014-04-04	2016-05-27	4	0.0110193	11	0.0303030	5	0.0137741	0	0.0000000
Honduras	2020_04	445,171	184	2009-07-02	2016-12-31	257,650	0.5789588	75,222	0.1690295	37,554	0.0843866	2,016	0.0045301
Indonesia	2020_04	1,116,769	81	2009-02-27	2016-12-31	48,224	0.0431929	9,184	0.0082259	228,673	0.2048160	7,912	0.0070866
Iran	2018_10	610,511	170	2010-12-26	2016-12-31	208,913	0.3422094	184,103	0.3015694	46,321	0.0758760	4,427	0.0072516
Iran	2019_01	974,971	1,159	2009-09-05	2016-12-31	779,790	0.8007702	626,645	0.6435048	54,180	0.0556377	16,370	0.0168104
Iran	2019_06	1,076,599	187	2008-04-30	2016-12-31	682,320	0.6345599	561,082	0.5218081	58,003	0.0539430	6,482	0.0060283
Russia	2018_10	7,427,397	3,251	2009-05-09	2016-12-31	14,938,741	2.0116371	10,659,464	1.4353936	1,163,029	0.1566124	558,878	0.0752580
Russia	2019_01	433,791	328	2010-08-03	2016-12-31	56,783	0.1316716	50,222	0.1164576	14,444	0.0334936	3,169	0.0073485
Serbia	2020_04	1,777,478	777	2009-07-15	2016-12-31	253,785	0.1428759	792,609	0.4462230	95,758	0.0539098	2,691	0.0015150
Venezuela	2019_01	6,345,692	655	2010-01-11	2016-12-31	5,500,667	0.8678140	909,629	0.1435078	472,708	0.0745769	64,493	0.0101748
Venezuela	2019_06	187,408	17	2012-04-25	2016-12-31	523,982	2.7964200	498,026	2.6578964	71,415	0.3811321	68,479	0.3654630

A2 Survey

The YouGov survey waves took place between April 2016 and November 2016. However, the survey question asking who respondents voted for was obtained in a post-election questionaire that was collected by YouGov separately after the election. The fielding dates of each survey wave were as follows:

- Wave 1: April 9–May 1 2016
- Wave 2: September 9–October 9 2016
- Wave 3: October 25–November 7 2016
- Post-election (whether a respondent voted, and for whom)

In Supplementary Table A2, we provide summary statistics regarding the socio-demographic characteristics of the YouGov survey Twitter users. For comparison, we also provide analogous data from a 2018 survey of Twitter users conducted by Pew Research Center.[2] Although our population of interest is US Twitter users and not the general US population, we also provide data from the 2016 American Community Survey (ACS) for comparison. These data show that our sample is nevertheless similar to the demographic profile of US adults. Supplementary Table A3 further provides the comparison by party ID of the sub-samples of respondents who use Twitter and who agreed to provide links to their accounts and the rest of the survey panel.

Supplementary Table A2: Comparison of YouGov Twitter users to US Census Bureau data and a Pew Research survey of Twitter users. Numbers indicate percentages.

Variable	Category	YouGov (2016)	Pew Twitter Survey (2018)	US Population (ACS, 2016)
sex	male	44.2	50	48.7
	female	55.8	50	51.3
age	18-29	12.7	29	21.5
	30-49	40.2	44	33.4
	50-64	33.2	19	25.4
	65+	13.8	8	19.7
education	no college	65.0	58	68.7
	college+	35.0	42	31.3
income	$< 30,000$	28.1	23	25.8
	30,000+	71.9	77	74.2
race	white	77.4	60	72.6
	poc	22.6	28	27.4

[2] https://www.pewresearch.org/internet/2019/04/24/sizing-up-twitter-users/

Supplementary Table A3: Partisan distribution of survey respondents broken down by Twitter use (respondents whose exposure we observe on Twitter vs. those who do not use Twitter/have not provided their data). Numbers indicate percentages.

	YouGov	
Party ID	With Twitter Data	Without Twitter Data
Strong Democrat	30.9	27.1
Moderate Democrat	13.8	12.5
Weak Democrat	10.6	7.7
Independent	12.8	17.1
Weak Republican	8.9	8.1
Moderate Republican	10.0	10.6
Strong Republican	11.6	14.5
N	1496.0	2004.0

A3 Survey questions

In Figure 4 of the main article, we estimate the relationship between exposure to posts from Russian foreign influence accounts and respondents' political ideology and issues positions. Below we present the question wording for each outcome that is presented in Figure 4. We then present the question text for the question that asks respondents how they rank the presidential comparisons, as used in Figure 5.

Political ideology

```
As shown on the scales below, some people in the US tend to identify
more with the political left, while others tend to identify more with the
political right.  And of course, some other people have opinions somewhere
in between.  Please place yourself on this scale.  Then place both of the
US's two major parties on the same scale.  Then, place each of the following
candidates for president on the same scale.

[0 = Far left, 1, 2, ..., 99, 100 = Far right]
```

Use of military force

```
As shown on the scales below, some people think that military force should
be used only as a last resort, while other people think that military force
is usually the best way to solve international problems.  And of course,
some other people have opinions somewhere in between.  Please place yourself
on this scale.  Then place each of the following national figures on the
same scale.

[0 = Military force should be used only as a last resort, 1, 2, ..., 99, 100
= Military force is usually the best way to solve international problems]
```

Chinese tariffs

```
As shown on the scale below, some people think that we should increase
tariffs on goods from China to protect American jobs from unfair
competition, others think that this would lead to a trade war that would
harm the American economy and cost jobs.  And of course some people have
opinions in between.  Please place yourself on this scale.  Then place each
of the following national figures on the same scale.

[0 = Increase Tariffs on China, 1, 2, ..., 99, 100 = A Trade War Would Cost
Jobs]
```

Free trade

As shown on the scales below, some people think that we should reduce trade with other countries to protect American jobs from foreign competition, while others believe that we should increase trade to benefit American consumers and create more markets for American goods. And of course others have opinions in between. Please place yourself on this scale. Then place each of the following national figures on the same scale.

[0 = Reduce free trade with other countries, 1, 2, ..., 99, 100 = Increase free trade with other countries]

Ban on Muslims

As shown on the scale below, some people think we should bar Muslims from entering the US to prevent terrorism, others think it is an essential aspect of the United States that we do not discriminate based on religion, and of course some people have opinions in between. Please place yourself on this scale. Then place each of the following national figures on the same scale.

[0 = Bar Muslims From Entering the US, 1, 2, ..., 99, 100 = Do Not Discriminate Based on Religion]

Expanding the ACA

The Affordable Care Act, signed into law by President Obama in 2010, restructured the US health care system. As shown on the scales below, some people think that the health care law should be repealed entirely, while others think it should be expanded to cover more people and services. And of course, some other people have opinions somewhere in between, such as simply keeping the law as it is now. Please place yourself on this scale. Then place each of the following national figures on the same scale.

[0 = Completely replease the entire health care law, 1, 2, ..., 99, 100 = Expand the health care law's coverage]

Obamacare

As shown on the scale below, some people think we should repeal Obamacare and start over to handle health insurance, others think we should leave

Obamacare in place, but expand coverage, and of course some people have opinions in between. Please place yourself on this scale. Then place each of the following national figures on the same scale.

[0 = Repeal Obamacare, 1, 2, ..., 99, 100 = Start Over]

Building a wall

As shown on the scale below, some people think we should build a wall between the United States and Mexico, while others think that this would be a foolish waste of resources and not address real issues of immigration. And of course some people have opinions in between. Please place yourself on this scale. Then place each of the following national figures on the same scale.

[0 = Build a wall, 1, 2, ..., 99, 100 = Address immigration issues via other means]

Immigration

As shown on the scales below, some people think that the US should deport all illegal immigrants and others think we should instead provide them with a path to citizenship. And of course others have opinions in between, such as allowing illegal immigrants to obtain guest worker status. Please place yourself on this scale. Then place each of the following national figures on the same scale.

[0 = Deport all illegal immigrants back to their home countries, 1, 2, ..., 99, 100 = Provide all illegal immigrants an eventual path to citizenship]

Presidential candidate rankings

Please rank how much you like each of the following candidates for president. We are not asking you who you will vote for, but which candidate would be your first choice to be president, which would be your next favorite choice to be president, and so on.

Hillary Clinton (D)
Bernie Sanders (D)
Donald Trump (R)

John Kasich (R)
Ted Cruz (R)

A4 Twitter data collection for YouGov survey

The lists of friends for all YouGov respondents who provided their Twitter profiles were retrieved in April 2016. After the election, in December 2016, we further collected the posts of YouGov respondents and their friends. In total we collected 1,244,124,493 tweets from the timelines of our respondents' friends. These tweets represent the corpus of posts that potentially appeared in respondents' timelines.

We then matched the posts appearing in the timelines of respondents' friends to the list of accounts identified as belonging to foreign influence campaigns by the Twitter Elections Integrity project, as described above. To do so, we extracted the unique identifiers of the authors of tweets that were posted or retweeted by respondents' friends. In addition to original tweets, we extracted the unique identifiers of the authors of retweets and quote tweets. These unique identifiers were matched to the unique identifiers of accounts linked to foreign influence campaigns.

Overall, we identified 786,634 posts from foreign influence campaign accounts that had the potential to appear in respondents' timelines. These posts could appear and be viewed by respondents, although given the black-box of the Twitter's timeline algorithm, this cannot be known with certainty for any specific post. As we note in the article, we follow the literature [e.g. 3] in referring to these posts as "exposures.' '

A5 Accounts of politicians and national news media organizations

In the Results section of the main article, we compare the number of exposures from Russian Internet Research Agency accounts to those from politicians and national news media. The list of politicians includes all Members of Congress and political candidates in the 2016 race. National news media include all major national news organizations, including their politics-related specialized accounts (e.g. ABC This Week, CBS 60 Minutes, New York Times The Upshot). Lists of these sets of actors are available on request.

B Equivalence testing for minimal relationships

In the article, we present a series of regression results in Figure 4 to examine the relationship between exposure to posts from Russian Internet Research Agency accounts and political attitudes and polarization. In general, we find no meaningful relationship between exposure and these outcomes. Theoretically, this is consistent with the descriptive data, which demonstrates that exposure to posts from Russian foreign influence accounts was overshadowed by posts from politicians and news media, and was heavily concentrated among those who identify as Republican partisans.

However, because the absence of statistically significant results is not necessarily strong evidence of a minimal relationship [8], in this section we examine these results in an equivalence testing framework [4, 7, 8]. This framework addresses the problem that the absence of statistically significant differences is not necessarily itself strong evidence of a negligible relationship. If a study is heavily underpowered, meaningful relationships will be difficult to detect—for lack of sufficient data—under the null hypothesis of no difference. We first briefly explain the use of equivalence testing, after which we present the results.

In the standard null hypothesis framework, a researcher typically seeks to find statistical evidence to reject the null hypothesis that a relationship is zero. In an equivalence testing framework, researchers seek to provide statistical evidence to reject the hypothesis that a relationship is meaningful. Thus, whereas in null hypothesis testing framework one seeks to find evidence against a null relationship, in equivalence testing one seeks to find evidence in favor of a negligible one.

Equivalence testing operates by constructing a lower bound ϵ_L and an upper bound ϵ_H that a researcher defines as the range of the magntiude of a relationship that substantively would be considered negligible (e.g. $\epsilon_L = -.25$, $\epsilon_H = .25$). Given this range, one then seeks to reject the hypothesis that the magnitude of the relationship is greater than $|\epsilon|$.[3] Statistically rejecting the null hypothesis that the relationship is of magnitude $|\epsilon|$ or larger helps provide evidence for the alternative hypothesis that the relationship is negligible. In practice, this typically works by applying a Two One-Sided Test (TOST) procedure that, as the name suggests, first calculates a one-sided test to provide evidence that a relationship is greater than the lower bound ϵ_L, and calculates a second one-sided test to provide evidence that the relationship is less than the upper bound ϵ_H. Jointly, these tests provide evidence that a relationship lies within the range $[\epsilon_L, \epsilon_H]$ that defines what would be considered a negligible.

In political science research, defining the values of ϵ_L and ϵ_H is typically done with outcome variables that are re-scaled in terms of standard deviations. For the issue-based outcomes, this is sensible because the scale (from 0-100) is arbitrary. Because vote choice is more comprehensible on a percentage point scale, we use simulation in the main article to examine the magnitude of the relationship between exposure and voting behavior. In standard deviations, defining what constitutes a negligible relationship is an area of active research. In a recent article, for example, Hartman and Hidalgo [4] define meaningful relationships as those greater than 0.36 standard deviations; others more conservatively, as those greater than

[3] Equivalence bounds do not need to be defined symmetrically—such that ϵ_L and ϵ_H can be of different magnitude—but this is rare in practice.

0.25 standard deviations [5, 6]; and Cohen [2], in his classic work, as less than 0.2 standard deviations. We include all three of these definitions of minimal relationships for comparison in our results below. Nevertheless, one can also simply calculate Two One-Sided Test intervals that define the range of the size of a relationship that would not be rejected by the Two One-Sided Test procedure. Defining ϵ_L and ϵ_H anywhere outside of this interval would, in a Two One-Sided Test procedure, lead to the rejection of the null hypothesis of a meaningful relationship. These intervals thus allow researchers to assess the strength of the evidence in favor of a minimal relationship, without reference to pre-defined values of ϵ_L and ϵ_H (which we nevertheless include for reference below).

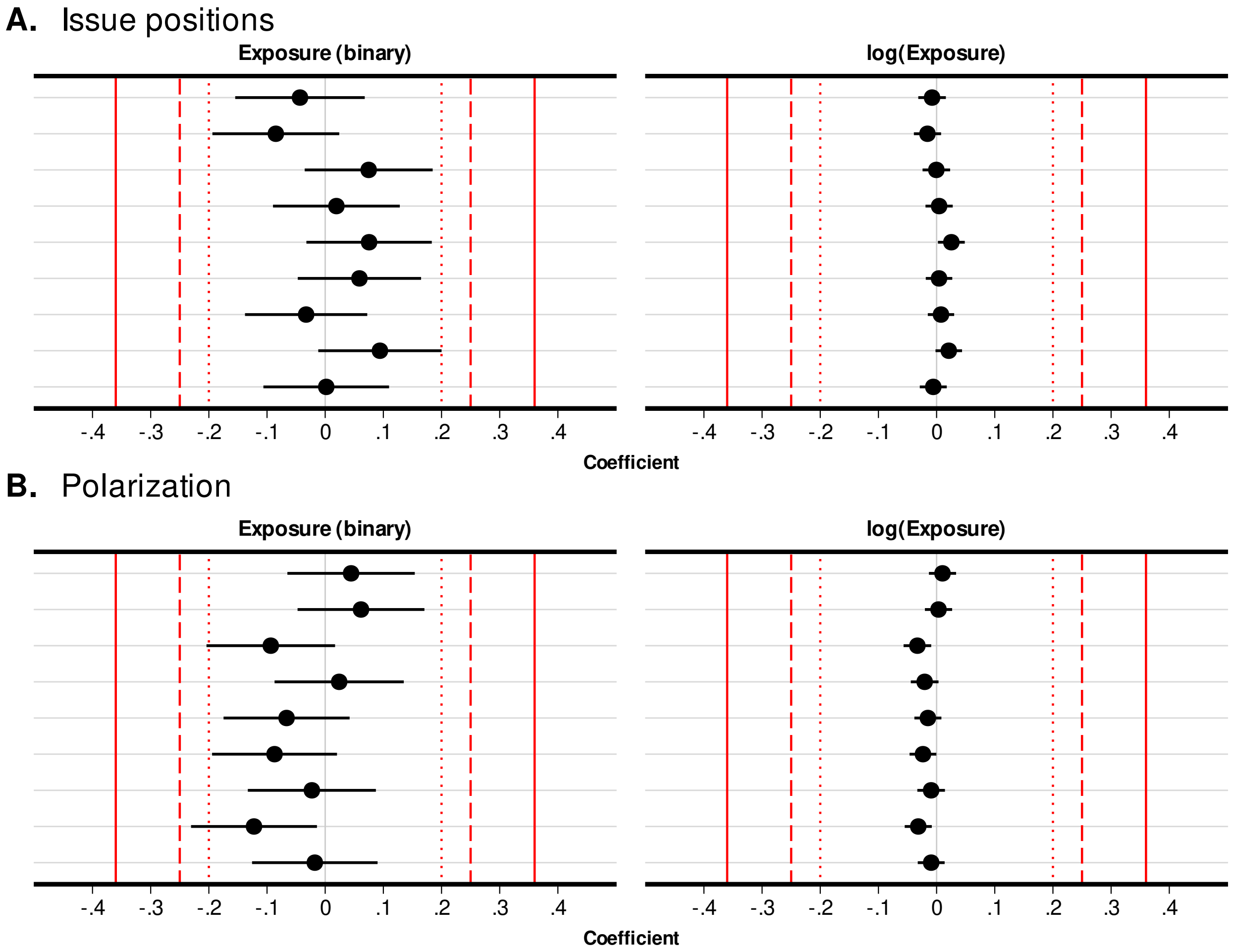

Supplementary Figure B3: Two One-Sided Test (TOST) 95% Intervals for the relationship between exposure to tweets from Russian foreign influence accounts and issue positions and polarization. Each point (•) represents the estimated relationships in standardized units; each horizontal line (—) represents a 95% TOST interval: the range of the size of a relationship that cannot be rejected by a Two One-Sided Test. The vertical lines (in red) represent what the literature generally considers minimal relationships: the solid line, that defined by Hartman and Hidalgo [4] ($|\epsilon| < 0.36$); the dashed line, that defined by Ho et al. [5] and Imbens and Rubin [6] ($|\epsilon| < 0.25$); and the dotted line, that defined by Cohen [2] ($|\epsilon| < 0.2$). $n = 1,496$ survey respondents

To apply equivalence tests to investigate the strength of the evidence as it relates to negligible relationships, we standardize each of the outcome variables and calculate TOST intervals for the magnitude of the relationship between exposure to social media posts from Russian foreign influence accounts. We do so for each of the outcomes in Figures 4 of the main article, and examine these intervals in relation to values of ϵ_L and ϵ_H defined in the literature.

The equivalence test results for estimates of the relationships between exposure to posts from Russian foreign influence accounts and each outcome in Figures 4 in the main article are presented in Supplementary Figure B3. The values of ϵ_L and ϵ_H that define the range of minimal relationships are shown as defined by Hartman and Hidalgo [4] (solid line), Ho et al. [5] and Imbens and Rubin [6] (dashed line), and Cohen [2] (dotted line). The first column of Panels A and B of Supplementary Figure B3 present estimates of the relationship between log(exposure + 1) and each issue position. Estimates appear relatively small primarily because exposure is measured on the log scale, thus indicating that the relationship between a one unit change in the (log) count of exposure to social media posts from Russian foreign influence accounts. The second column presents exposure measured as a binary variable, as is typically used in the literature on equivalence testing. By all definitions of minimal relationships (as defined by the vertical lines in each figure), estimates for all issues positions, and all but one measure of polarization are consist with negligible relations. We can also examine this differently, by averaging across all issue position outcomes, in which we find that the magnitude of the estimated relationship between exposure to posts from Russian influence accounts and issues positions is a mere 6% of a standard deviation, and for polarization, 5% of a standard deviation.

C Exposure to foreign influence accounts

C1 Overall

We calculate exposure to foreign influence accounts by extracting all posts that contain tweets, retweets, and quote tweets from accounts that were identified by Twitter as belonging to state-backed foreign influence campaigns, and were in the collected feeds from respondents' friends.

Overall, 1,042 respondents (64%) were exposed at least once to posts from accounts associated with foreign influence campaigns from Russia, China, Iran, or Venezuela. For overall exposure, we treat the presence of tweets, retweets, and quote tweets in the timelines of respondents' friends as indicators of potential exposure. Among respondents, 946 (63%) were exposed to at least one post from a Russian Internet Research Agency account, making it by far the most wide-reaching foreign influence campaign during the 2016 US presidential election.

In terms of direct exposure to foreign influence accounts (i.e. following the identified accounts themselves), 112 (7% of our sample) respondents followed at least one account associated with a foreign influence campaign from Russia, China, Iran, or Venezuela. Among respondents, 51 (3.4%) followed an account associated with the Russian campaign specifically.

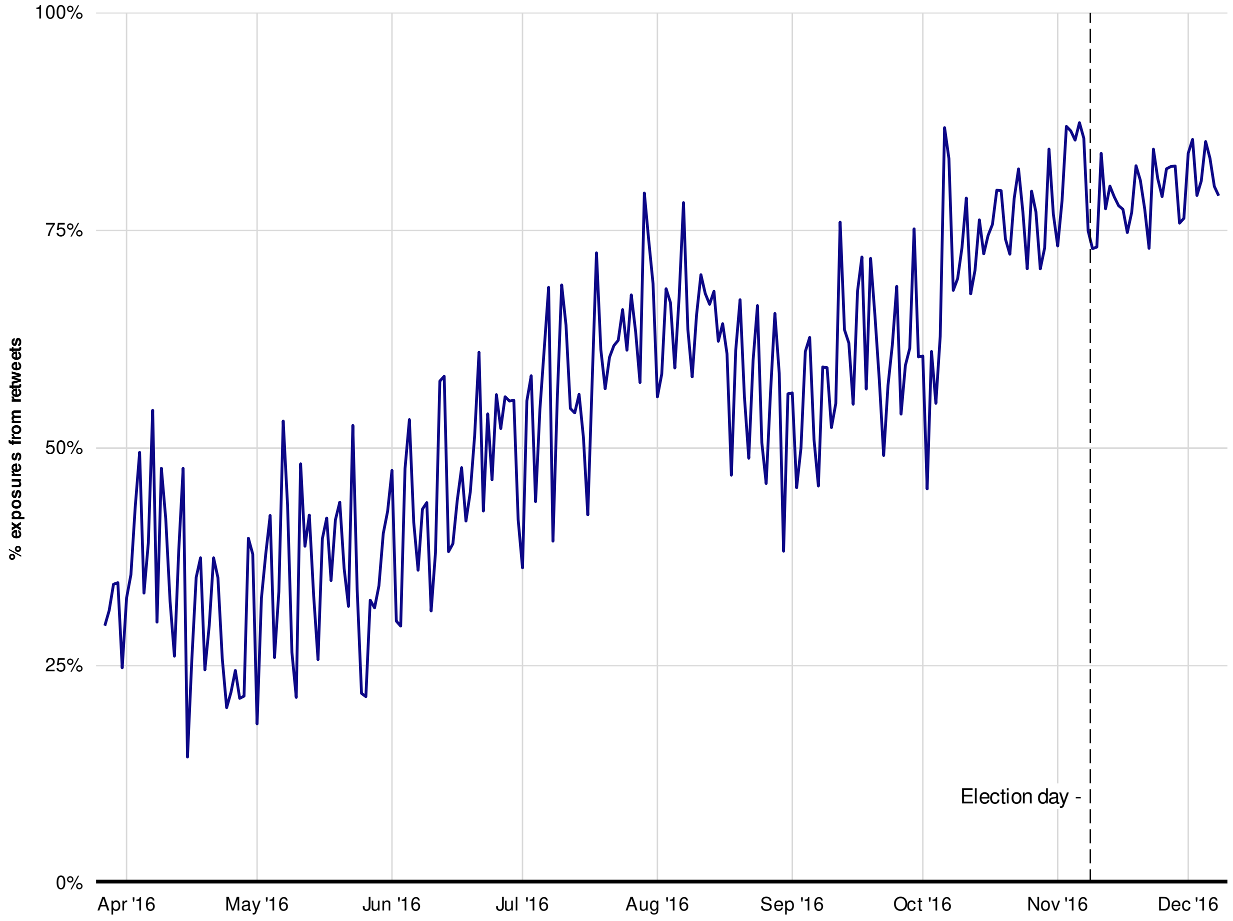

Supplementary Figure C4: Percentage of exposures to retweets from the Russian disinformation campaign

C2 Party ID

In the main article, we examine the relationship between the self-identified party ID of respondents and their exposure of posts from Russian Internet Research Agency accounts. The model presented in Panel B of Figure 3, however, models a linear relationship between party ID and exposure to posts from Russian foreign influence accounts. Thus, although there is a clear and strong relationship between being a self-identified Republican and exposure to Russian foreign influence accounts, it may be the case that this relationship is U-shaped: that exposure was higher both among those who strongly identify as Republicans and Democrats. Theoretically, this might be expected if the goals of the Russian foreign influence campaign was to further alienate those who strongly identify with the Democratic Party (e.g. Bernie Sanders supporters) from their party's establishment nominee. If this were the goal, we might expect high levels of exposure to Russian electoral interference among strongly identifying Democrats. Descriptively, the data in Panel A of Figure 3 suggest that this is not the case. We test this more formally in a regression framework by fitting an equivalent model to that in Panel B of Figure 3, but including a squared party ID term in the model. This term captures the theoretical expectation that both those who strongly identify as Republicans and Democrats were similarly exposed to posts from Russia's foreign influence campaign.

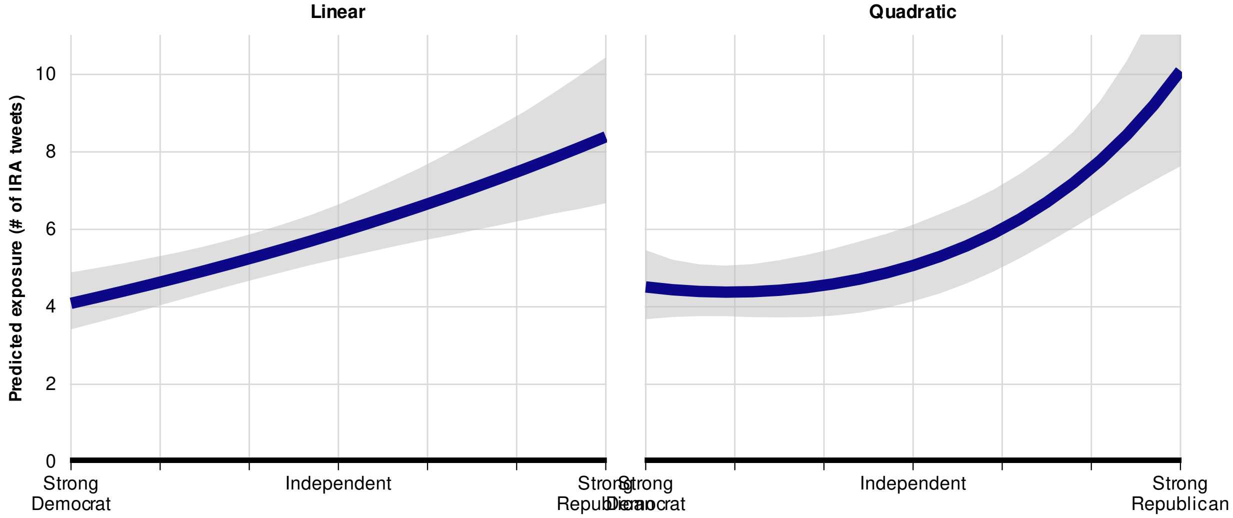

Supplementary Figure C5: Relationship between party ID and exposure to posts from Russian Internet Research Agency (IRA) accounts. Predicted values with 95% prediction intervals are shown

Regression results from both the model in the main manuscript (Panel B of Figure 3), and the model with the squared term for party ID are presented in Supplementary Table C4. We test whether the inclusion of the squared term increases model fit by conducting a likelihood ratio test that compares the model with the squared term to the model without it. It does not ($p = 0.09$). We nevertheless graph the predicted relationship between party ID and $log(exposure + 1)$ for both models to examine the shape of the relationship. Results are presented in Supplementary Figure C5. As the figure shows, the relationship between party ID and respondents' level of exposure to posts from Russian foreign influence accounts is similarly increasing as one identifies more strongly as a Republican. Importantly, in the

second panel of Supplementary Figure C5, we do not observe a U-shape relationship to the extent that strongly identifying Democrats were not more likely to be exposed to more posts from the Russian foreign influence campaign than weakly identifying Democrats or independents. To test the sensitivity of these results to modelling assumptions we also fit a quasi-Poisson regression models with the indepedent variables. The results of theses models are shown in Supplementary Table C5.

Supplementary Table C4: Regression models for the relationship between party ID and (log) exposure to posts from Russian Internet Research Agency accounts

	log(Exposure + 1)			
	(1)	(2)	(3)	(4)
Party ID	0.616***	−0.300	0.673***	0.771***
	(0.169)	(0.574)	(0.170)	(0.128)
Party ID^2		1.001		
		(0.599)		
Age	0.011**	0.010*	0.011*	0.023***
	(0.004)	(0.004)	(0.004)	(0.003)
Woman	−0.350**	−0.349**	−0.349**	−0.302***
	(0.121)	(0.121)	(0.121)	(0.091)
College-educated	−0.159	−0.155	−0.150	−0.004
	(0.125)	(0.125)	(0.125)	(0.094)
Income	−0.077	−0.084	−0.076	−0.077
	(0.066)	(0.066)	(0.065)	(0.049)
Person of Color	0.054	0.053		
	(0.147)	(0.147)		
Other/Hispanic			−0.217	−0.030
			(0.184)	(0.139)
Black			0.400	0.398**
			(0.204)	(0.153)
Region: Northeast	−0.030	−0.031	−0.016	−0.022
	(0.168)	(0.168)	(0.168)	(0.126)
Region: Midwest	−0.033	−0.027	−0.028	−0.136
	(0.153)	(0.153)	(0.152)	(0.114)
Region: West	0.087	0.094	0.136	0.008
	(0.162)	(0.162)	(0.163)	(0.123)
Freq. of social media use	0.184***	0.180**	0.183***	0.048
	(0.056)	(0.056)	(0.055)	(0.042)
log(Total Tweets)				0.701***
				(0.021)
Constant	0.319	0.473	0.309	−6.112***
	(0.479)	(0.488)	(0.479)	(0.409)
N	1,400	1,400	1,400	1,400

*p < .05; **p < .01; ***p < .001

Party ID is a 7-category variable, which is rescaled to range from 0 to 1, where 0 indicates "Strong Democrat", 1 indicates "Strong Republican.", and the mid-point, 0.5, indicates "Independent."

Supplementary Table C5: Quasi-Poisson regression models for the relationship between party ID and (log) exposure to posts from Russian Internet Research Agency accounts

	Exposure			
	(1)	(2)	(3)	(4)
Party ID	2.391***	0.057	2.428***	2.324***
	(0.542)	(2.077)	(0.582)	(0.263)
Party ID^2		2.162		
		(1.890)		
Age	0.048***	0.047***	0.049***	0.040***
	(0.013)	(0.014)	(0.014)	(0.008)
Woman	0.528	0.515	0.531	0.432*
	(0.374)	(0.379)	(0.397)	(0.180)
College-educated	0.220	0.211	0.220	0.660***
	(0.375)	(0.379)	(0.398)	(0.166)
Income	0.132	0.122	0.133	−0.050
	(0.186)	(0.187)	(0.198)	(0.086)
Person of Color	−0.808	−0.844		
	(0.790)	(0.801)		
Other/Hispanic			−1.110	−0.808
			(1.123)	(0.476)
Black			−0.268	−0.398
			(1.227)	(0.503)
Region: Northeast	0.344	0.362	0.345	0.442
	(0.511)	(0.519)	(0.542)	(0.231)
Region: Midwest	0.354	0.374	0.352	−0.519*
	(0.451)	(0.457)	(0.478)	(0.227)
Region: West	0.422	0.452	0.427	−0.024
	(0.471)	(0.479)	(0.500)	(0.207)
Freq. of social media use	0.780*	0.770*	0.781*	0.555***
	(0.376)	(0.377)	(0.398)	(0.107)
log(Total Tweets)				1.473***
				(0.082)
Constant	−3.972	−3.477	−4.019	−18.111***
	(2.858)	(2.892)	(3.030)	(1.326)
N	1,400	1,400	1,400	1,400

*p < .05; **p < .01; ***p < .001

Party ID is a 7-category variable, which is rescaled to range from 0 to 1, where 0 indicates "Strong Democrat", 1 indicates "Strong Republican.", and the mid-point, 0.5, indicates "Independent."

C3 Alternative model specification (Poisson)

In Panel A of Figure 3 in the main article, we present the average of the number of exposures by respondents to Russian foreign influence account tweets throughout the campaign, broken down by the partisanship of the respondent. In Panel B of Figure 3, we present analogous results in a regression framework, including a number of respondent covariates. In Panel B of Figure 3, the regression outcome is defined as $log(exposure + 1)$, where exposure is the count of posts in a respondent's feed. Because the outcome is a count variable, we also model the relationship using a quasi-poisson regression. The results, shown in Supplementary Figure C6 show similar results to those in Figure 3 of the main article.

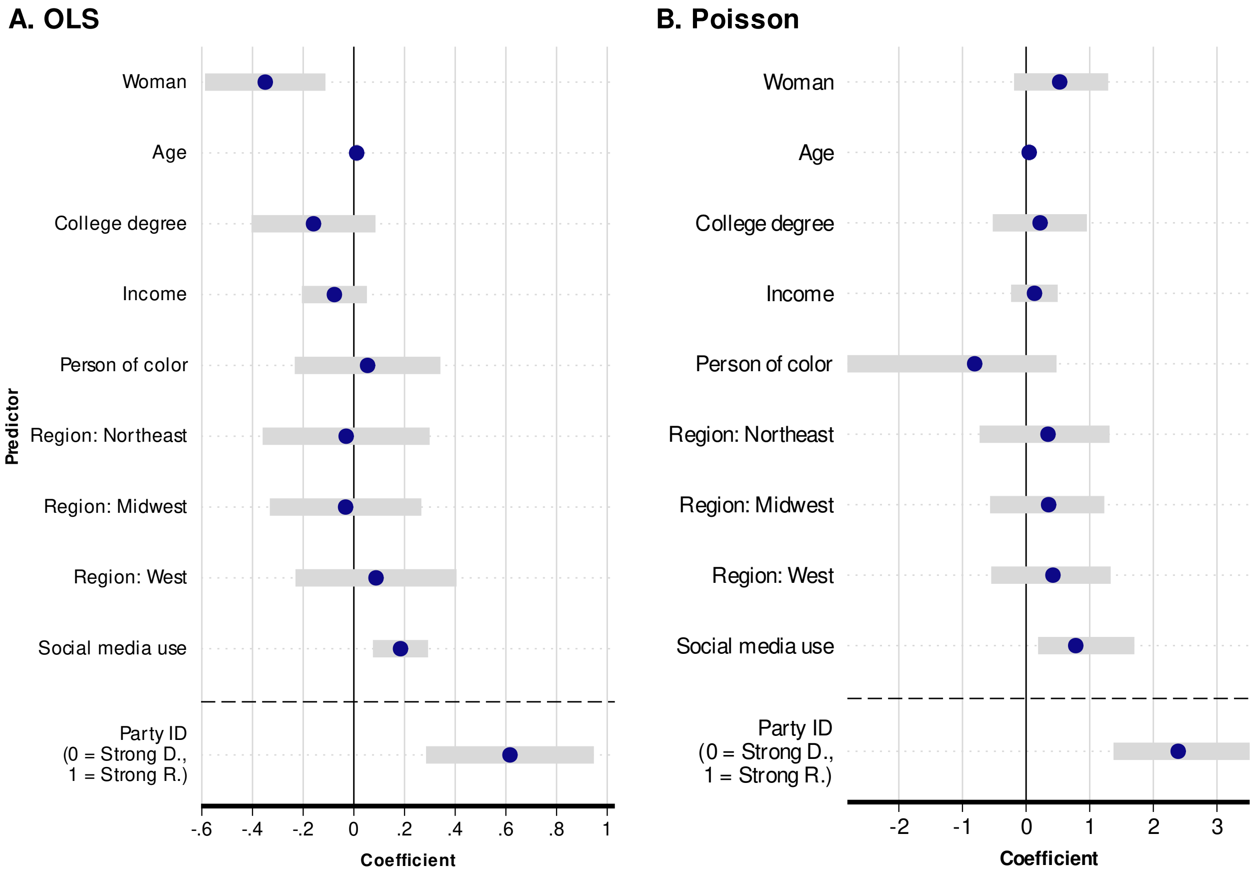

Supplementary Figure C6: Partisanship and exposure to posts from Russian Internet Research Agency accounts during the 2016 US election campaign (quasi-poisson regression). Error bars represent 95% confidence intervals around the point estimate of the regression coefficient. $n = 1,496$ survey respondents

D Exposure to political actors

Analogous to Panels B and C in Figure 1 in the main article, we calculate and visualize the empirical cumulative distributions of exposure to tweets by survey respondents from news media organizations (Panel A of Supplementary Figure D7) and politicians (Panel C of Supplementary Figure D7). Panels B and D present the empirical cumulative distributions of tweets sent by media and politicians' accounts, respectively. As the figure shows, exposure to politicians and news media is less concentrated among small groups of users compared to exposure to tweets from Russian foreign influence accounts. As noted in the article, just 1% of respondents account for 70% of exposures of Russian foreign influence accounts. Among news media organizations and politicians, 1% of respondents account of 24% and 37% of exposures.

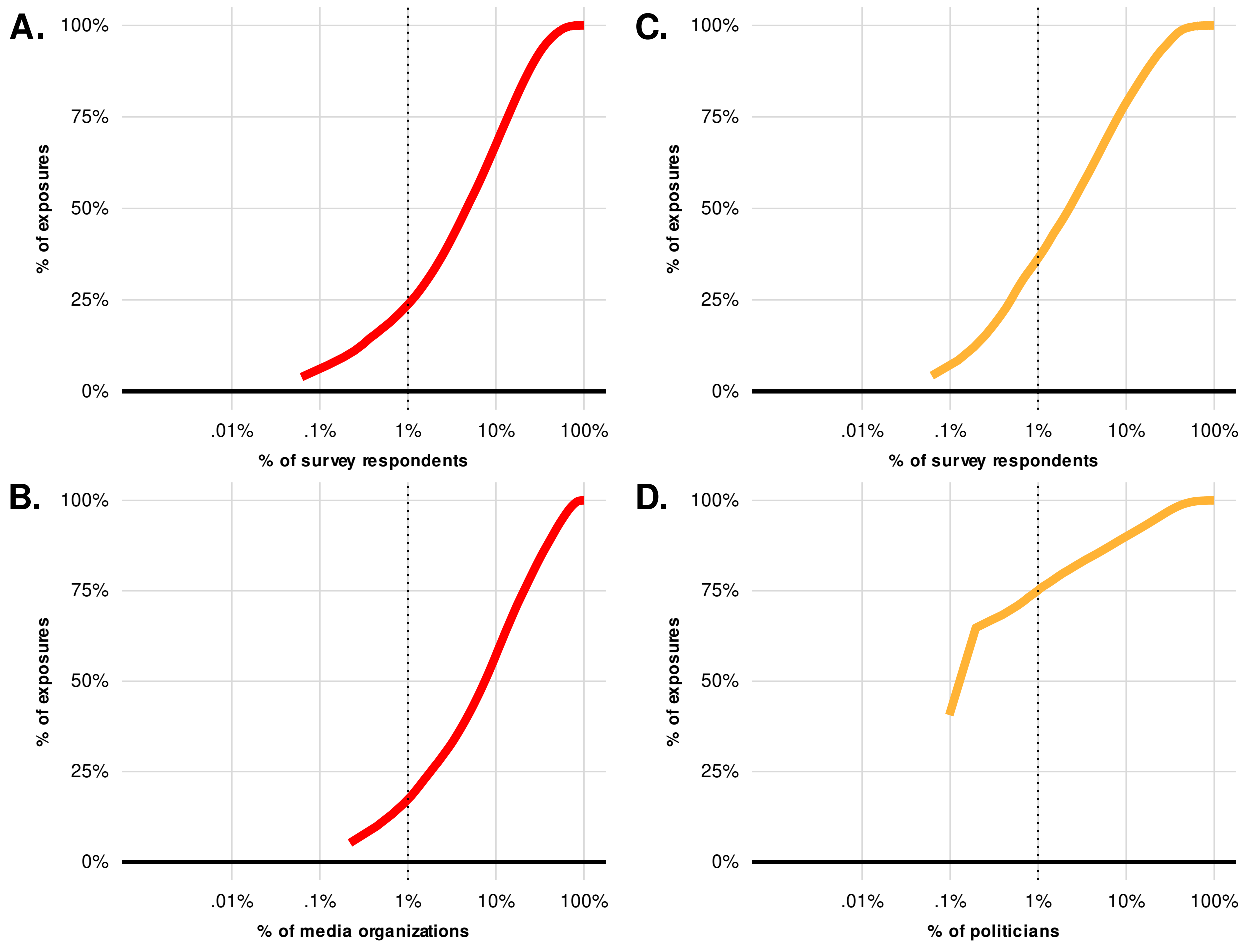

Supplementary Figure D7: Partisanship and exposure to posts from news media and politicians accounts during the 2016 US election campaign

E Vote change regression models

E1 Details of response variables coding

To measure changes in candidate preferences between the first and last waves of the survey, we construct three outcomes that use respondents' rankings of Hillary Clinton and Donald Trump in wave 1 as a baseline:

(1) vote choice (from baseline to voting for one of the two main candidates),

(2) vote choice (from baseline to voting for one of the two main candidates, a 3rd party candidate or abstaining from voting),

(3) rank (from baseline to rankings of Hillary Clinton and Donald Trump).

All three outcome variables are constructed in such a way that positive values indicate changes in the direction favorable to Donald Trump. More formally, we measure the changes between the first wave w_1 and the last wave w_j: $\Delta preference_{iw_j-iw_1}$, such that:

$$\Delta preference_{iw_j-iw_1} = Preference^{(.)}_{iw_j} - Preference_{iw_1}$$

with the candidate preference in the first wave in each case measured as:

$$Preference_{iw_1} = 1(TrumpRank_{iw_1} < ClintonRank_{iw_1})$$

where $ClintonRank_{iw_1}$ and $TrumpRank_{iw_1}$ are respondent i's ranks of Hillary Clinton and Donald Trump in the first wave. Note that the lower the assigned rank, the more preferred a given candidate is. In other words, $1(TrumpRank_{iw_1} < ClintonRank_{iw_1})$ is an indicator function that takes the value of 1 when Donald Trump is preferred to Hillary Clinton by respondent i in wave 1, and 0 otherwise. The three comparison measures in the last wave $Preference^{(.)}_{iw_j}$ are calculated as follows:

$$Preference^{(1)}_{iw_4} = 1(TrumpVote_{iw_4} = 1)$$

$$Preference^{(2)}_{iw_4} = 1(Non - ClintonVote_{iw_4} = 1)$$

$$Preference^{(3)}_{iw_3} = 1(TrumpRank_{iw_3} < ClintonRank_{iw_3})$$

where $TrumpVote_{iw_4}$ is a post-election measure of voting for Donald Trump, $Non - ClintonVote_{iw_4}$ is a post-election measure of voting for Donald Trump, other candidates or abstaining from voting, $ClintonRank_{iw_3}$ and $TrumpRank_{iw_3}$ are the respondent i's ranks of Hillary Clinton and Donald Trump in the third wave.

E2 Regression models using the count of exposures

Supplementary Tables E6, E7 and E8 show the full output for linear models used in the main text of the article.

Supplementary Table E6: Regression models for the relationship between exposure to tweets from Russian Internet Research Agency accounts and a change in ranking a vote for Trump over that for Clinton

	(1)	(2)	(3)
log(Exposure + 1)	−0.002	−0.003	−0.003
	(0.004)	(0.004)	(0.005)
Age	0.0002	0.0001	0.0001
	(0.001)	(0.001)	(0.001)
Woman	0.035	0.035	0.035
	(0.018)	(0.018)	(0.018)
College-educated	0.007	0.008	0.008
	(0.019)	(0.019)	(0.019)
Income	−0.011	−0.012	−0.012
	(0.010)	(0.010)	(0.010)
Person of Color	−0.013		
	(0.021)		
Other/Hispanic		−0.044	−0.044
		(0.026)	(0.026)
Black		0.023	0.023
		(0.030)	(0.030)
Region: Northeast	0.001	0.004	0.004
	(0.028)	(0.028)	(0.028)
Region: Midwest	−0.022	−0.021	−0.021
	(0.021)	(0.021)	(0.021)
Region: West	−0.012	−0.007	−0.007
	(0.026)	(0.026)	(0.026)
Freq. of social media use	−0.004	−0.004	−0.004
	(0.007)	(0.007)	(0.008)
log(Total Tweets)			−0.0001
			(0.005)
Party ID	−0.002	0.005	0.005
	(0.024)	(0.024)	(0.024)
Constant	0.052	0.051	0.052
	(0.065)	(0.065)	(0.075)

$^{*}p < .05$; $^{**}p < .01$; $^{***}p < .001$

Supplementary Table E7: Regression models for the relationship between exposure to tweets from Russian Internet Research Agency accounts and change in election-day vote for Trump

	(1)	(2)	(3)
log(Exposure + 1)	−0.001	−0.002	0.012
	(0.003)	(0.006)	(0.008)
Age	−0.0002	−0.001	−0.002
	(0.0005)	(0.001)	(0.001)
Woman	0.035*	0.093**	0.096***
	(0.015)	(0.028)	(0.028)
College-educated	0.012	0.045	0.042
	(0.015)	(0.029)	(0.029)
Income	−0.004	−0.034*	−0.034*
	(0.008)	(0.015)	(0.015)
Person of Color	0.025		
	(0.021)		
Other/Hispanic		0.048	0.048
		(0.052)	(0.052)
Black		0.077	0.071
		(0.043)	(0.042)
Region: Northeast	−0.004	−0.022	−0.022
	(0.022)	(0.041)	(0.041)
Region: Midwest	−0.003	0.016	0.019
	(0.018)	(0.037)	(0.037)
Region: West	0.009	−0.009	−0.008
	(0.024)	(0.040)	(0.040)
Freq. of social media use	0.005	0.007	0.009
	(0.006)	(0.014)	(0.014)
log(Total Tweets)			−0.023*
			(0.010)
Party ID	0.027	−0.055	−0.069
	(0.021)	(0.037)	(0.038)
Constant	−0.043	0.066	0.276*
	(0.055)	(0.119)	(0.141)

*p < .05; **p < .01; ***p < .001

Supplementary Table E8: Regression models for the relationship between exposure to tweets from Russian Internet Research Agency accounts and a change in election-day vote (Trump, 3rd party, did not vote) over a vote for Clinton

	(1)	(2)	(3)
log(Exposure + 1)	−0.002	−0.002	0.012
	(0.006)	(0.006)	(0.008)
Age	−0.001	−0.001	−0.002
	(0.001)	(0.001)	(0.001)
Woman	0.093**	0.093**	0.096***
	(0.028)	(0.028)	(0.028)
College-educated	0.045	0.045	0.042
	(0.029)	(0.029)	(0.029)
Income	−0.034*	−0.034*	−0.034*
	(0.015)	(0.015)	(0.015)
Person of Color	0.061		
	(0.037)		
Other/Hispanic		0.048	0.048
		(0.052)	(0.052)
Black		0.077	0.071
		(0.043)	(0.042)
Region: Northeast	−0.023	−0.022	−0.022
	(0.041)	(0.041)	(0.041)
Region: Midwest	0.015	0.016	0.019
	(0.037)	(0.037)	(0.037)
Region: West	−0.011	−0.009	−0.008
	(0.040)	(0.040)	(0.040)
Freq. of social media use	0.007	0.007	0.009
	(0.014)	(0.014)	(0.014)
log(Total Tweets)			−0.023*
			(0.010)
Party ID	−0.057	−0.055	−0.069
	(0.037)	(0.037)	(0.038)
Constant	0.067	0.066	0.276*
	(0.119)	(0.119)	(0.141)

*p < .05; **p < .01; ***p < .001

E3 Regression models using a binary measure of exposure

Supplementary Tables E9, E10 and E11 show the full output for linear models used in the main text of the article.

Supplementary Table E9: Regression models for the relationship between exposure to at least 1 tweet from Russian Internet Research Agency accounts and a change in ranking a vote for Trump over that for Clinton

	(1)	(2)	(3)
1(Exposure > 0)	−0.034	−0.034	−0.046
	(0.020)	(0.020)	(0.025)
Age	0.0001	0.00002	0.0001
	(0.001)	(0.001)	(0.001)
Woman	0.034	0.034	0.034
	(0.018)	(0.018)	(0.018)
College-educated	0.007	0.008	0.009
	(0.019)	(0.019)	(0.019)
Income	−0.011	−0.011	−0.011
	(0.009)	(0.009)	(0.009)
Person of Color	−0.013		
	(0.021)		
Other/Hispanic		−0.043	−0.043
		(0.026)	(0.026)
Black		0.022	0.023
		(0.030)	(0.030)
Region: Northeast	−0.002	0.0001	−0.001
	(0.028)	(0.028)	(0.029)
Region: Midwest	−0.022	−0.020	−0.021
	(0.021)	(0.021)	(0.021)
Region: West	−0.013	−0.008	−0.009
	(0.025)	(0.025)	(0.026)
Freq. of social media use	−0.002	−0.002	−0.003
	(0.007)	(0.007)	(0.007)
log(Total Tweets)			0.004
			(0.005)
Party ID	−0.006	0.001	0.002
	(0.023)	(0.023)	(0.023)
Constant	0.067	0.066	0.031
	(0.066)	(0.066)	(0.072)

*p < .05; **p < .01; ***p < .001

Supplementary Table E10: Regression models for the relationship between exposure to at least 1 tweet from Russian Internet Research Agency accounts and change in election-day vote for Trump

	(1)	(2)	(3)
1(Exposure > 0)	−0.024	−0.024	−0.031
	(0.016)	(0.016)	(0.018)
Age	−0.0003	−0.0003	−0.0002
	(0.0005)	(0.0005)	(0.0005)
Woman	0.034*	0.034*	0.034*
	(0.015)	(0.015)	(0.015)
College-educated	0.012	0.012	0.012
	(0.015)	(0.015)	(0.015)
Income	−0.004	−0.004	−0.004
	(0.008)	(0.008)	(0.008)
Person of Color	0.025		
	(0.021)		
Other/Hispanic		0.038	0.038
		(0.030)	(0.030)
Black		0.012	0.012
		(0.026)	(0.026)
Region: Northeast	−0.007	−0.007	−0.008
	(0.022)	(0.022)	(0.023)
Region: Midwest	−0.004	−0.004	−0.004
	(0.018)	(0.018)	(0.018)
Region: West	0.008	0.007	0.006
	(0.024)	(0.024)	(0.024)
Freq. of social media use	0.006	0.006	0.006
	(0.006)	(0.006)	(0.006)
log(Total Tweets)			0.003
			(0.005)
Party ID	0.025	0.023	0.023
	(0.020)	(0.020)	(0.020)
Constant	−0.028	−0.027	−0.047
	(0.056)	(0.055)	(0.064)

*p < .05; **p < .01; ***p < .001

Supplementary Table E11: Regression models for the relationship between exposure to at least 1 tweet from Russian Internet Research Agency accounts and a change in election-day vote (Trump, 3rd party, did not vote) over a vote for Clinton

	(1)	(2)	(3)
1(Exposure > 0)	−0.027	−0.027	0.016
	(0.030)	(0.030)	(0.038)
Age	−0.001	−0.001	−0.001
	(0.001)	(0.001)	(0.001)
Woman	0.091**	0.091**	0.093***
	(0.028)	(0.028)	(0.028)
College-educated	0.045	0.045	0.042
	(0.029)	(0.029)	(0.029)
Income	−0.034*	−0.034*	−0.035*
	(0.015)	(0.015)	(0.015)
Person of Color	0.062		
	(0.037)		
Other/Hispanic		0.049	0.048
		(0.052)	(0.052)
Black		0.077	0.075
		(0.043)	(0.043)
Region: Northeast	−0.026	−0.025	−0.021
	(0.041)	(0.041)	(0.041)
Region: Midwest	0.015	0.015	0.018
	(0.037)	(0.037)	(0.037)
Region: West	−0.012	−0.010	−0.007
	(0.040)	(0.040)	(0.041)
Freq. of social media use	0.008	0.008	0.009
	(0.014)	(0.014)	(0.014)
log(Total Tweets)			−0.016
			(0.009)
Party ID	−0.059	−0.057	−0.059
	(0.036)	(0.037)	(0.037)
Constant	0.080	0.080	0.211
	(0.121)	(0.121)	(0.134)

*p < .05; **p < .01; ***p < .001

E4 Regression models using following Russian Internet Research Agency accounts as a variable of interest

In the article, we present results of models estimating the relationship between exposure to posts from Russian Internet Research Agency accounts and voting preferences and behavior, and similarly, the relationship between exposure to at least one such post on the same outcomes. In this section we also present results from a regression model examining the relationship between following a Russian Internet Research Agency account and these vote-based outcomes. Unlike exposure, which as we discuss in the Results section was primarily incidental, following an Internet Research Agency account is directly chosen by respondents. Nevertheless, it is useful to examine whether there is a relationship between following and voting behavior. Results analogous to those in Figure 5 of the main article for following a Russian Internet Research Agency account are presented in Supplementary Figure E8. Similar to the results regarding exposure, we find no evidence that following a Russian Internet Research Agency account is associated with changes in any of the measured voting preferences and behavior. Supplementary Tables E12, E13 and E14 show the full output of these linear models.

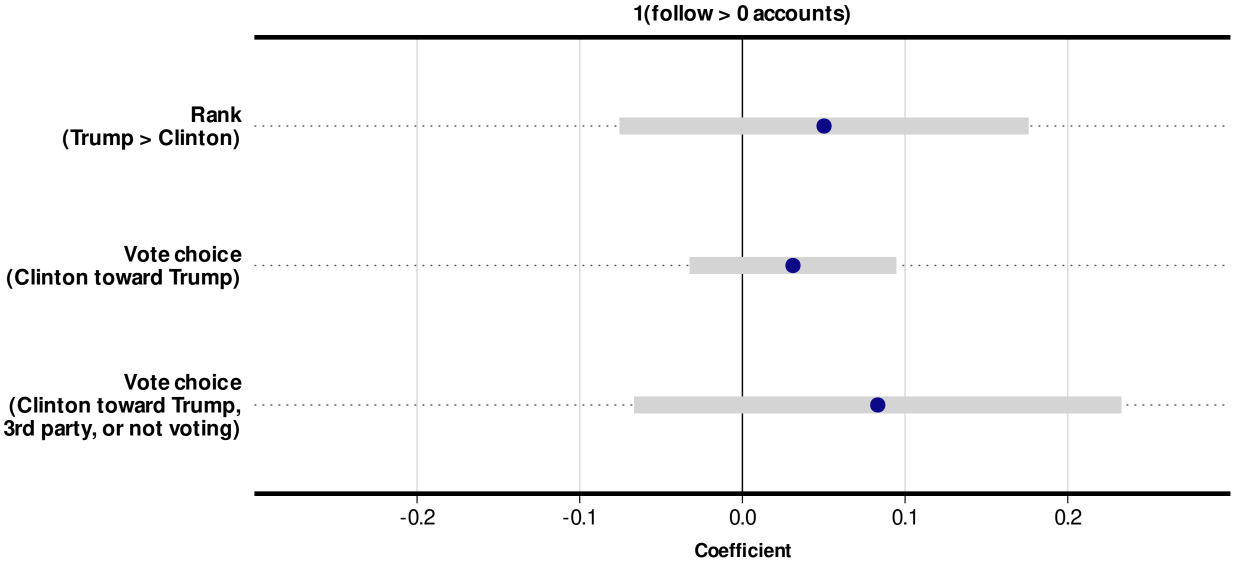

Supplementary Figure E8: Regression results of the relationship between following a Russian Internet Research Agency (IRA) account and voting behavior. Error bars represent 95% confidence intervals around point estimates of the regression coefficients. $n = 1,496$ survey respondents

Supplementary Table E12: Regression models of the relationship between following at least 1 Russian Internet Research Agency (IRA) account account and a change in ranking a vote for Trump over that for Clinton

	(1)	(2)	(3)
1(Follow > 0 IRA account)	0.050	0.052	0.059
	(0.064)	(0.064)	(0.063)
Age	0.0002	0.0001	0.00005
	(0.001)	(0.001)	(0.001)
Woman	0.036*	0.036*	0.036*
	(0.018)	(0.018)	(0.018)
College-educated	0.009	0.010	0.009
	(0.019)	(0.019)	(0.019)
Income	−0.012	−0.012	−0.012
	(0.009)	(0.009)	(0.009)
Person of Color	−0.013		
	(0.021)		
Other/Hispanic		−0.043	−0.044
		(0.026)	(0.026)
Black		0.023	0.023
		(0.030)	(0.030)
Region: Northeast	0.0001	0.002	0.002
	(0.028)	(0.028)	(0.028)
Region: Midwest	−0.022	−0.021	−0.020
	(0.021)	(0.021)	(0.021)
Region: West	−0.012	−0.007	−0.006
	(0.025)	(0.025)	(0.026)
Freq. of social media use	−0.004	−0.005	−0.004
	(0.007)	(0.007)	(0.008)
log(Total Tweets)			−0.003
			(0.004)
Party ID	−0.005	0.002	0.001
	(0.023)	(0.023)	(0.023)
Constant	0.052	0.051	0.079
	(0.065)	(0.065)	(0.074)

*p < .05; **p < .01; ***p < .001

Supplementary Table E13: Regression models of the relationship between following at least 1 Russian Internet Research Agency (IRA) account and change in election-day vote for Trump

	(1)	(2)	(3)
1(Follow > 0 IRA account)	0.031	0.030	0.036
	(0.032)	(0.033)	(0.034)
Age	−0.0002	−0.0002	−0.0003
	(0.0005)	(0.0005)	(0.0005)
Woman	0.036*	0.036*	0.036*
	(0.015)	(0.015)	(0.015)
College-educated	0.013	0.013	0.013
	(0.015)	(0.015)	(0.015)
Income	−0.004	−0.004	−0.004
	(0.008)	(0.008)	(0.008)
Person of Color	0.025		
	(0.021)		
Other/Hispanic		0.038	0.038
		(0.031)	(0.031)
Black		0.012	0.012
		(0.026)	(0.026)
Region: Northeast	−0.004	−0.005	−0.005
	(0.022)	(0.022)	(0.022)
Region: Midwest	−0.002	−0.002	−0.002
	(0.018)	(0.018)	(0.018)
Region: West	0.010	0.008	0.008
	(0.024)	(0.024)	(0.024)
Freq. of social media use	0.005	0.005	0.005
	(0.006)	(0.006)	(0.006)
log(Total Tweets)			−0.003
			(0.005)
Party ID	0.024	0.022	0.022
	(0.020)	(0.020)	(0.020)
Constant	−0.042	−0.042	−0.017
	(0.055)	(0.055)	(0.064)

*p < .05; **p < .01; ***p < .001

Supplementary Table E14: Regression models of the relationship between following at least 1 Russian Internet Research Agency (IRA) account and a change in election-day vote (Trump, 3rd party, did not vote) over a vote for Clinton

	(1)	(2)	(3)
1(Follow > 0 IRA account)	0.083	0.084	0.124
	(0.076)	(0.076)	(0.077)
Age	−0.001	−0.001	−0.001
	(0.001)	(0.001)	(0.001)
Woman	0.094***	0.094***	0.093***
	(0.028)	(0.028)	(0.028)
College-educated	0.046	0.046	0.043
	(0.029)	(0.029)	(0.029)
Income	−0.034*	−0.034*	−0.034*
	(0.015)	(0.015)	(0.015)
Person of Color	0.062		
	(0.037)		
Other/Hispanic		0.048	0.049
		(0.052)	(0.052)
Black		0.078	0.078
		(0.043)	(0.043)
Region: Northeast	−0.025	−0.024	−0.026
	(0.041)	(0.041)	(0.041)
Region: Midwest	0.016	0.016	0.018
	(0.036)	(0.036)	(0.036)
Region: West	−0.011	−0.009	−0.007
	(0.040)	(0.040)	(0.040)
Freq. of social media use	0.006	0.006	0.008
	(0.014)	(0.014)	(0.014)
log(Total Tweets)			−0.016*
			(0.007)
Party ID	−0.060	−0.058	−0.062
	(0.037)	(0.037)	(0.037)
Constant	0.070	0.069	0.227
	(0.119)	(0.119)	(0.135)

*p < .05; **p < .01; ***p < .001

E5 Alternative coding of race

For ease of comparison in Supplementary Figure E9 we present a subset of the models from Supplementary Tables E6-E11 above, which is analogous to Figure 5 of the main text, but has an alternative coding of control variable race. Here, rather than treating all respondents of color similarly, we break them further down into Black and Hispanic/Other groups. Consistent with the results in the main manuscript, we find no evidence of a relationship between exposure and switches in voting preferences.

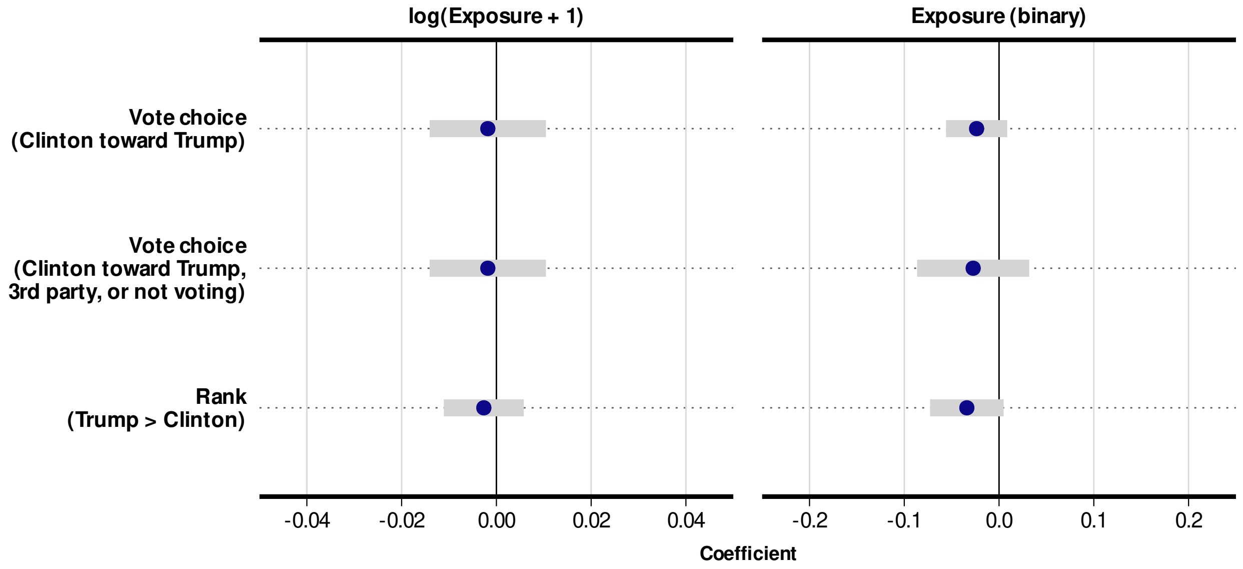

Supplementary Figure E9: Regression results of exposure to Russian Internet Research Agency accounts and changes in issue-based and ideological positioning with alternative race break-down. Error bars represent 95% confidence intervals around point estimates of the regression coefficients. $n = 1,496$ survey respondents

E6 Alternative measure of overall Twitter activity

As another robustness check, rather than relying on a self-reported measure of Twitter activity, that might make respondents less likely to be exposed to tweets from Russian foreign influence campaigns, we control for the total number of tweets posted by their friends. In constructing this measure we rely on our own collected data to estimate the number of potential exposures to other tweets (non-Internet Research Agency) in respondents' timelines. These models are also presented in Supplementary Tables E6-E11 above, but for ease of comparison to Figure 5 of the main manuscript in Supplementary Figure E10 we present a subset of them that use log(Total Tweets) as one of control variables. Consistent with the results in the main manuscript, we find no evidence of a relationship between exposure and switches in voting preferences.

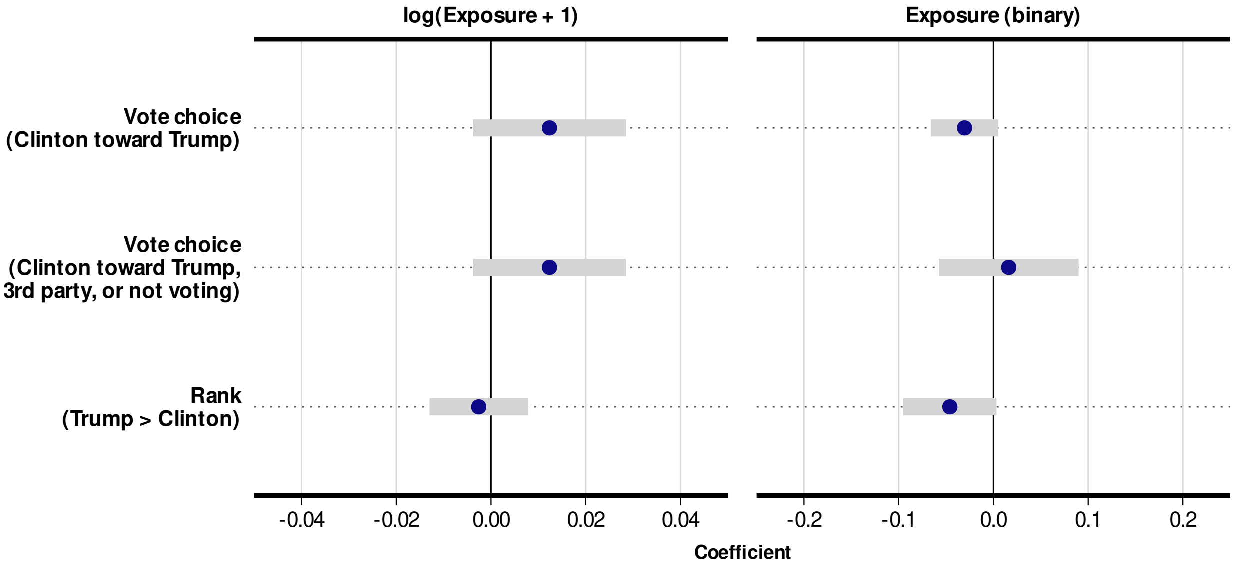

Supplementary Figure E10: Regression results of exposure to Russian Internet Research Agency accounts and changes in issue-based and ideological positioning with alternative measure of overall potential exposures on Twitter. Error bars represent 95% confidence intervals around point estimates of the regression coefficients. $n = 1,496$ survey respondents

E7 Final vote preference as outcome

In Supplementary Table E15, we provide results for models in which vote choice in the final wave of the survey is the outcome variable, and a respondent's wave 1 preference is used as a control. This differs from the approach in the main manuscript, in which the outcome variable is a change score (change in vote preference from wave 1 to the final wave). However, Allison [1] shows that controlling for a previous measured outcome rather than using change scores can result in more biased estimates if the previous measured outcome is correlated with the error term.

Supplementary Table E15: Regression models for the relationship between exposure to tweets from Russian Internet Research Agency (IRA) accounts and ranking a vote for Trump over that for Clinton in W3

	Vote rank(Trump>Clinton)			Vote choice(Clinton→Trump)			Vote choice(Clinton→Trump/3rd/Not)		
	(1)	(2)	(3)	(4)	(5)	(6)	(7)	(8)	(9)
log(Exposure + 1)	0.002			0.003			0.012*		
	(0.004)			(0.003)			(0.005)		
1(Exposure > 0)		−0.027			−0.012			0.006	
		(0.018)			(0.015)			(0.024)	
1(Follow > 0 IRA account)			0.062			0.034			0.113
			(0.056)			(0.025)			(0.059)
Age	0.001	0.001	0.001	0.001	0.001	0.001	0.001	0.002	0.001
	(0.001)	(0.001)	(0.001)	(0.0005)	(0.0005)	(0.0005)	(0.001)	(0.001)	(0.001)
Woman	0.016	0.014	0.016	0.027*	0.025	0.027*	0.047*	0.043	0.044*
	(0.017)	(0.017)	(0.017)	(0.014)	(0.014)	(0.014)	(0.022)	(0.022)	(0.022)
College-educated	−0.013	−0.014	−0.013	−0.001	−0.003	−0.002	−0.019	−0.020	−0.019
	(0.018)	(0.018)	(0.018)	(0.014)	(0.014)	(0.014)	(0.023)	(0.023)	(0.023)
Income	−0.014	−0.014	−0.015	−0.009	−0.010	−0.009	−0.040***	−0.041***	−0.041***
	(0.009)	(0.009)	(0.009)	(0.008)	(0.008)	(0.007)	(0.012)	(0.012)	(0.012)
People of Color	−0.034	−0.033	−0.033	0.004	0.005	0.005	−0.010	−0.007	−0.005
	(0.020)	(0.020)	(0.020)	(0.019)	(0.019)	(0.019)	(0.029)	(0.029)	(0.029)
Region: Northeast	0.023	0.019	0.021	−0.0005	−0.003	−0.002	0.011	0.010	0.006
	(0.026)	(0.026)	(0.026)	(0.020)	(0.020)	(0.020)	(0.033)	(0.033)	(0.033)
Region: Midwest	−0.012	−0.012	−0.012	0.005	0.003	0.004	0.023	0.022	0.022
	(0.019)	(0.019)	(0.019)	(0.017)	(0.017)	(0.017)	(0.029)	(0.029)	(0.029)
Region: West	−0.008	−0.008	−0.007	0.012	0.011	0.012	0.001	0.002	0.002
	(0.024)	(0.024)	(0.024)	(0.021)	(0.021)	(0.021)	(0.031)	(0.032)	(0.031)
Freq. of social media use	−0.003	−0.001	−0.003	0.007	0.008	0.007	0.008	0.009	0.009
	(0.007)	(0.007)	(0.007)	(0.006)	(0.006)	(0.006)	(0.010)	(0.010)	(0.010)
Party ID	0.265***	0.263***	0.264***	0.315***	0.313***	0.314***	0.706***	0.707***	0.705***
	(0.045)	(0.045)	(0.045)	(0.053)	(0.053)	(0.053)	(0.049)	(0.049)	(0.049)
W1 Rank Trump over Clinton	0.711***	0.713***	0.711***	0.704***	0.708***	0.707***	0.154***	0.161***	0.161***
	(0.036)	(0.036)	(0.036)	(0.042)	(0.042)	(0.042)	(0.040)	(0.040)	(0.040)
Constant	0.025	0.040	0.029	−0.070	−0.057	−0.064	0.021	0.026	0.034
	(0.064)	(0.065)	(0.064)	(0.053)	(0.054)	(0.054)	(0.089)	(0.091)	(0.091)

$^{*}p < .05$; $^{**}p < .01$; $^{***}p < .001$

F Linear models of ideological positioning

F1 Details of outcome variable coding

To measure changes in ideological positioning between the first and final waves of the survey, we create two types of outcome variables: relative issue position and perceived polariztion of the candidates on different policy issues (measured on a 0-100 scale).

For the first measure we calculate how far from Hillary Clinton a respondent is relative to Donald Trump. In order to calculate this, we take the absolute difference between a respondent's own position on a given issue and the position that they perceive each of the two candidates holds, such that positive values indicate more proximity to Donald Trump and negative values indicate more proximity to Hillary Clinton. Afterwards, we take the difference between the last wave and the first wave such that positive values reflect moves towards Trump's position on a given issue and negative values indicate moves towards Clinton's position on a given issue. More precisely, for changes in relative issue position placement we measure $\Delta position_{iw_3-iw_1}$, such that:

$$\Delta position_{iw_3-iw_1} = RelativePosition_{iw_3} - RelativePosition_{iw_1}$$

The values of $RelativePosition_{iw_1}$ and $RelativePosition_{iw_3}$ are measured as follows:

$$RelativePosition_{iw_1} = |Position_{iw_1} - ClintonPosition_{iw_1}| - |Position_{iw_1} - TrumpPosition_{iw_1}|$$

$$RelativePosition_{iw_3} = |Position_{iw_3} - ClintonPosition_{iw_3}| - |Position_{iw_3} - TrumpPosition_{iw_3}|$$

where $Position_{iw_j}$ is the self-placement of a respondent i in wave j and $ClintonPosition_{iw_j}$ and $TrumpPosition_{iw_j}$ are the respondent's placements of Hillary Clinton and Donald Trump on the same issue.

In a similar way, we calculate the changes in perceived polarization between the two candidates from the first to last wave of the survey. However, rather than measuring how far away from a given candidate a respondent places herself, we measure how far she perceives Hillary Clinton to be from Donald Trump on a policy issue. More formally, for changes in perceived polarization we measure $\Delta polarization_{iw_3-iw_1}$, such that:

$$\Delta polarization_{iw_3-iw_1} = Polarization_{iw_3} - Polarization_{iw_1}$$

The values of $Polarization_{iw_1}$ and $Polarization_{iw_3}$ are measured as follows:

$$Polarization_{iw_1} = |ClintonPosition_{iw_1} - TrumpPosition_{iw_1}|$$

$$Polarization_{iw_3} = |ClintonPosition_{iw_3} - TrumpPosition_{iw_3}|$$

where $ClintonPosition_{iw_j}$ and $TrumpPosition_{iw_j}$ are respondent i's placements of Hillary Clinton and Donald Trump on the same issue scale in wave j.

F2 Relative self-placement

Supplementary Tables F16-F24 show the complete output of issue-based and ideological positioning models presented in Figure 4A of the main text.

Supplementary Table F16: Regression models of the relationship between exposure to Russian Internet Research Agency (IRA) accounts and political ideology

	**Issue-based scale (political ideology)**								
	(1)	(2)	(3)	(4)	(5)	(6)	(7)	(8)	(9)
log(Exposure + 1)	−0.224	−0.239	0.264						
	(0.376)	(0.374)	(0.513)						
1(Exposure > 0)				−1.244	−1.257	0.724			
				(2.078)	(2.074)	(2.331)			
1(Follow > 0 IRA account)							−1.163	−1.008	0.483
							(3.355)	(3.344)	(3.473)
Age	0.052	0.049	0.028	0.047	0.043	0.033	0.052	0.048	0.033
	(0.061)	(0.061)	(0.065)	(0.062)	(0.062)	(0.062)	(0.061)	(0.061)	(0.063)
Woman	−1.535	−1.555	−1.490	−1.532	−1.546	−1.529	−1.474	−1.485	−1.553
	(1.863)	(1.857)	(1.854)	(1.866)	(1.861)	(1.858)	(1.848)	(1.843)	(1.842)
College-educated	−0.126	−0.068	−0.180	−0.100	−0.040	−0.195	−0.111	−0.049	−0.171
	(1.949)	(1.946)	(1.953)	(1.941)	(1.938)	(1.949)	(1.947)	(1.944)	(1.955)
Income	0.038	0.050	0.076	0.051	0.064	0.061	0.053	0.066	0.061
	(1.037)	(1.035)	(1.037)	(1.037)	(1.035)	(1.035)	(1.038)	(1.036)	(1.036)
People of Color	0.020			0.023			0.002		
	(2.716)			(2.714)			(2.718)		
Other/Hispanic		−2.545	−2.653		−2.522	−2.660		−2.528	−2.641
		(3.013)	(3.027)		(3.012)	(3.024)		(3.014)	(3.023)
Black		3.514	3.350		3.489	3.395		3.450	3.423
		(4.567)	(4.610)		(4.568)	(4.593)		(4.581)	(4.587)
Region: Northeast	3.539	3.741	3.807	3.450	3.652	3.838	3.604	3.804	3.755
	(2.952)	(2.936)	(2.929)	(2.935)	(2.920)	(2.908)	(2.970)	(2.952)	(2.948)
Region: Midwest	3.265	3.417	3.631	3.304	3.458	3.576	3.283	3.437	3.570
	(2.166)	(2.177)	(2.170)	(2.162)	(2.173)	(2.166)	(2.167)	(2.178)	(2.169)
Region: West	−1.724	−1.210	−1.043	−1.771	−1.264	−1.014	−1.774	−1.268	−1.042
	(2.419)	(2.443)	(2.450)	(2.423)	(2.449)	(2.448)	(2.425)	(2.452)	(2.455)
Freq. of social media use	0.783	0.788	0.851	0.816	0.819	0.839	0.755	0.756	0.860
	(0.784)	(0.786)	(0.790)	(0.797)	(0.799)	(0.797)	(0.776)	(0.778)	(0.791)
log(Total Tweets)			−0.794			−0.713			−0.624
			(0.705)			(0.606)			(0.537)
Party ID	12.815***	13.430***	12.948***	12.607***	13.205***	13.185***	12.682***	13.273***	13.159***
	(2.611)	(2.589)	(2.613)	(2.562)	(2.548)	(2.542)	(2.578)	(2.562)	(2.553)
Constant	−13.249	−13.550*	−6.358	−12.767	−13.069	−7.321	−13.453*	−13.753*	−7.775
	(6.782)	(6.828)	(9.283)	(6.853)	(6.898)	(8.379)	(6.805)	(6.849)	(8.396)

*p < .05; **p < .01; ***p < .001

Supplementary Table F17: Regression models of the relationship between exposure to posts from Russian Internet Research Agency (IRA) accounts and support for immigration

	Immigration								
	(1)	(2)	(3)	(4)	(5)	(6)	(7)	(8)	(9)
log(Exposure + 1)	−0.207	−0.213	0.347						
	(0.486)	(0.488)	(0.745)						
1(Exposure > 0)				0.065	0.052	2.953			
				(2.536)	(2.540)	(3.430)			
1(Follow > 0 IRA account)							6.881	6.919	8.842
							(5.746)	(5.746)	(5.921)
Age	0.031	0.030	0.007	0.030	0.030	0.015	0.026	0.025	0.005
	(0.076)	(0.076)	(0.078)	(0.077)	(0.077)	(0.077)	(0.075)	(0.075)	(0.076)
Woman	−2.978	−2.983	−2.905	−2.895	−2.898	−2.864	−2.784	−2.786	−2.878
	(2.240)	(2.239)	(2.223)	(2.225)	(2.224)	(2.209)	(2.233)	(2.232)	(2.235)
College-educated	−0.170	−0.164	−0.290	−0.148	−0.141	−0.347	−0.078	−0.070	−0.219
	(2.407)	(2.407)	(2.406)	(2.401)	(2.401)	(2.400)	(2.399)	(2.399)	(2.408)
Income	−0.525	−0.524	−0.497	−0.518	−0.517	−0.516	−0.546	−0.545	−0.545
	(1.260)	(1.260)	(1.256)	(1.260)	(1.260)	(1.260)	(1.258)	(1.259)	(1.260)
People of Color	−4.928			−4.945			−4.906		
	(2.750)			(2.751)			(2.753)		
Other/Hispanic		−5.572	−5.659		−5.554	−5.699		−5.564	−5.688
		(3.186)	(3.187)		(3.186)	(3.186)		(3.190)	(3.194)
Black		−3.987	−4.179		−4.057	−4.178		−3.946	−3.928
		(4.517)	(4.428)		(4.521)	(4.439)		(4.518)	(4.473)
Region: Northeast	3.247	3.298	3.347	3.289	3.337	3.552	3.153	3.205	3.132
	(3.384)	(3.394)	(3.394)	(3.405)	(3.414)	(3.443)	(3.375)	(3.385)	(3.375)
Region: Midwest	1.196	1.219	1.413	1.225	1.247	1.390	1.301	1.325	1.458
	(2.843)	(2.840)	(2.858)	(2.834)	(2.832)	(2.849)	(2.833)	(2.832)	(2.829)
Region: West	−2.493	−2.372	−2.193	−2.519	−2.406	−2.076	−2.444	−2.322	−2.053
	(3.004)	(3.008)	(3.044)	(3.010)	(3.013)	(3.065)	(3.012)	(3.018)	(3.047)
Freq. of social media use	−0.750	−0.750	−0.683	−0.789	−0.789	−0.759	−0.838	−0.839	−0.705
	(0.899)	(0.900)	(0.912)	(0.908)	(0.908)	(0.914)	(0.892)	(0.893)	(0.911)
log(Total Tweets)			−0.881			−1.049			−0.805
			(0.930)			(0.851)			(0.657)
Party ID	11.131***	11.278***	10.728**	10.991***	11.125***	11.050***	10.764***	10.908***	10.718***
	(3.200)	(3.236)	(3.266)	(3.190)	(3.221)	(3.214)	(3.179)	(3.212)	(3.205)
Constant	3.682	3.632	11.662	3.522	3.478	11.953	3.891	3.838	11.575
	(8.092)	(8.102)	(11.355)	(8.256)	(8.263)	(10.327)	(8.076)	(8.087)	(9.986)

*p < .05; **p < .01; ***p < .001

Supplementary Table F18: Regression models of the relationship between exposure to posts from Russian Internet Research Agency (IRA) accounts and support for banning Muslim immigrants

	**Muslim ban**								
	(1)	(2)	(3)	(4)	(5)	(6)	(7)	(8)	(9)
log(Exposure + 1)	0.943	0.958	1.160						
	(0.547)	(0.546)	(0.741)						
1(Exposure > 0)				2.805	2.821	2.426			
				(2.357)	(2.353)	(3.110)			
1(Follow > 0 IRA account)							12.660	12.574	11.951
							(7.044)	(7.026)	(7.282)
Age	0.010	0.013	0.004	0.025	0.028	0.030	0.008	0.010	0.016
	(0.087)	(0.088)	(0.090)	(0.087)	(0.088)	(0.089)	(0.087)	(0.088)	(0.089)
Woman	−4.267	−4.259	−4.230	−4.437	−4.433	−4.438	−4.399	−4.398	−4.372
	(2.361)	(2.360)	(2.358)	(2.342)	(2.341)	(2.339)	(2.352)	(2.352)	(2.355)
College-educated	−0.566	−0.584	−0.629	−0.664	−0.684	−0.654	−0.560	−0.578	−0.528
	(2.414)	(2.412)	(2.409)	(2.416)	(2.414)	(2.405)	(2.405)	(2.403)	(2.407)
Income	−2.188	−2.194	−2.185	−2.230	−2.236	−2.236	−2.261	−2.266	−2.265
	(1.195)	(1.193)	(1.194)	(1.197)	(1.196)	(1.195)	(1.191)	(1.190)	(1.189)
People of Color	−4.529			−4.502			−4.404		
	(3.196)			(3.192)			(3.185)		
Other/Hispanic		−2.762	−2.799		−2.854	−2.828		−2.863	−2.815
		(4.037)	(4.040)		(4.050)	(4.065)		(4.026)	(4.037)
Black		−7.029	−7.112		−6.832	−6.811		−6.585	−6.569
		(4.580)	(4.616)		(4.556)	(4.561)		(4.562)	(4.558)
Region: Northeast	8.026*	7.896*	7.914*	8.127*	8.005*	7.974*	7.584*	7.470*	7.496*
	(3.456)	(3.433)	(3.431)	(3.457)	(3.434)	(3.420)	(3.450)	(3.429)	(3.432)
Region: Midwest	4.808	4.750	4.833	4.681	4.625	4.600	4.812	4.759	4.704
	(3.049)	(3.050)	(3.039)	(3.052)	(3.054)	(3.041)	(3.048)	(3.050)	(3.038)
Region: West	1.625	1.292	1.355	1.816	1.509	1.460	1.849	1.561	1.471
	(2.982)	(3.009)	(3.007)	(2.986)	(3.014)	(3.004)	(2.983)	(3.009)	(2.994)
Freq. of social media use	−0.181	−0.179	−0.155	−0.180	−0.177	−0.181	−0.127	−0.123	−0.164
	(1.143)	(1.144)	(1.146)	(1.138)	(1.138)	(1.138)	(1.141)	(1.141)	(1.143)
log(Total Tweets)			−0.318			0.143			0.258
			(0.688)			(0.669)			(0.525)
Party ID	12.489***	12.077***	11.878***	13.252***	12.879***	12.889***	12.739***	12.393***	12.452***
	(3.257)	(3.255)	(3.299)	(3.268)	(3.268)	(3.267)	(3.267)	(3.267)	(3.276)
Constant	−1.291	−1.166	1.727	−2.144	−2.027	−3.180	−0.038	0.076	−2.416
	(10.042)	(10.043)	(11.905)	(10.154)	(10.153)	(11.457)	(10.005)	(10.002)	(11.326)

*p < .05; **p < .01; ***p < .001

Supplementary Table F19: Regression models of the relationship between exposure to posts from Russian Internet Research Agency (IRA) accounts and support for building a wall on the border with Mexico

	**Building a wall**								
	(1)	(2)	(3)	(4)	(5)	(6)	(7)	(8)	(9)
log(Exposure + 1)	0.839	0.854	0.902						
	(0.568)	(0.566)	(0.794)						
1(Exposure > 0)				3.768	3.790	3.709			
				(2.680)	(2.675)	(3.486)			
1(Follow > 0 IRA account)							8.452	8.365	7.406
							(6.018)	(6.004)	(6.168)
Age	−0.069	−0.067	−0.069	−0.051	−0.049	−0.048	−0.070	−0.068	−0.058
	(0.081)	(0.081)	(0.084)	(0.082)	(0.082)	(0.082)	(0.081)	(0.081)	(0.083)
Woman	3.316	3.320	3.326	3.237	3.237	3.237	3.143	3.140	3.192
	(2.453)	(2.452)	(2.456)	(2.438)	(2.438)	(2.438)	(2.424)	(2.424)	(2.429)
College-educated	−1.119	−1.139	−1.149	−1.203	−1.224	−1.218	−1.109	−1.130	−1.062
	(2.576)	(2.576)	(2.569)	(2.579)	(2.578)	(2.564)	(2.585)	(2.584)	(2.575)
Income	1.268	1.263	1.266	1.227	1.222	1.222	1.211	1.206	1.206
	(1.360)	(1.359)	(1.361)	(1.359)	(1.359)	(1.359)	(1.361)	(1.360)	(1.358)
People of Color	−10.511***			−10.488***			−10.406***		
	(2.994)			(3.000)			(3.006)		
Other/Hispanic		−8.818*	−8.828*		−8.883*	−8.878*		−8.902*	−8.825*
		(3.625)	(3.629)		(3.641)	(3.655)		(3.646)	(3.648)
Black		−12.920**	−12.939**		−12.771**	−12.767**		−12.547**	−12.531**
		(4.398)	(4.414)		(4.405)	(4.413)		(4.416)	(4.423)
Region: Northeast	6.547	6.428	6.432	6.738	6.624	6.618	6.222	6.114	6.158
	(3.441)	(3.446)	(3.452)	(3.458)	(3.464)	(3.480)	(3.434)	(3.438)	(3.430)
Region: Midwest	−1.726	−1.779	−1.761	−1.849	−1.901	−1.905	−1.738	−1.788	−1.861
	(3.123)	(3.120)	(3.136)	(3.112)	(3.110)	(3.118)	(3.110)	(3.109)	(3.108)
Region: West	3.536	3.219	3.234	3.682	3.384	3.375	3.728	3.448	3.307
	(3.331)	(3.312)	(3.306)	(3.344)	(3.327)	(3.320)	(3.342)	(3.324)	(3.293)
Freq. of social media use	−1.173	−1.172	−1.167	−1.234	−1.231	−1.232	−1.100	−1.096	−1.159
	(1.294)	(1.292)	(1.293)	(1.292)	(1.290)	(1.291)	(1.284)	(1.282)	(1.290)
log(Total Tweets)			−0.075			0.029			0.399
			(0.904)			(0.843)			(0.666)
Party ID	12.162**	11.764**	11.718**	12.864***	12.497***	12.499***	12.495***	12.153***	12.237***
	(3.721)	(3.737)	(3.765)	(3.645)	(3.668)	(3.669)	(3.657)	(3.674)	(3.667)
Constant	−0.223	−0.107	0.579	−1.669	−1.561	−1.799	0.707	0.815	−3.024
	(11.163)	(11.145)	(13.538)	(11.353)	(11.340)	(12.713)	(11.144)	(11.125)	(12.598)

*p < .05; **p < .01; ***p < .001

Supplementary Table F20: Regression models of the relationship between exposure to posts from Russian Internet Research Agency (IRA) accounts and support for Obamacare

	**Obamacare**								
	(1)	(2)	(3)	(4)	(5)	(6)	(7)	(8)	(9)
log(Exposure + 1)	0.305	0.264	0.791						
	(0.480)	(0.480)	(0.702)						
1(Exposure > 0)				−1.325	−1.394	−0.913			
				(2.613)	(2.613)	(3.579)			
1(Follow > 0 IRA account)							0.249	0.518	1.268
							(4.732)	(4.736)	(4.914)
Age	0.046	0.041	0.019	0.044	0.038	0.035	0.048	0.042	0.034
	(0.092)	(0.092)	(0.094)	(0.092)	(0.092)	(0.092)	(0.092)	(0.092)	(0.093)
Woman	0.040	0.010	0.076	−0.146	−0.167	−0.163	−0.062	−0.073	−0.106
	(2.464)	(2.458)	(2.461)	(2.450)	(2.445)	(2.445)	(2.457)	(2.452)	(2.452)
College-educated	−3.836	−3.765	−3.873	−3.861	−3.785	−3.818	−3.861	−3.783	−3.835
	(2.590)	(2.581)	(2.570)	(2.590)	(2.580)	(2.569)	(2.592)	(2.583)	(2.575)
Income	−1.910	−1.899	−1.873	−1.915	−1.903	−1.903	−1.920	−1.909	−1.907
	(1.291)	(1.286)	(1.285)	(1.289)	(1.285)	(1.285)	(1.290)	(1.285)	(1.285)
People of Color	1.168			1.179			1.179		
	(3.083)			(3.082)			(3.085)		
Other/Hispanic		−3.181	−3.294		−3.232	−3.265		−3.215	−3.286
		(3.988)	(3.988)		(3.984)	(3.980)		(3.987)	(3.988)
Black		7.152	6.942		7.246	7.221		7.230	7.215
		(4.248)	(4.263)		(4.248)	(4.253)		(4.256)	(4.258)
Region: Northeast	3.523	3.823	3.871	3.341	3.646	3.684	3.465	3.769	3.739
	(3.625)	(3.598)	(3.614)	(3.647)	(3.621)	(3.654)	(3.629)	(3.600)	(3.600)
Region: Midwest	5.787	5.940	6.147*	5.746	5.907	5.936	5.747	5.911	5.971
	(3.093)	(3.083)	(3.098)	(3.088)	(3.077)	(3.093)	(3.088)	(3.078)	(3.079)
Region: West	−0.044	0.810	0.987	0.039	0.896	0.959	0.032	0.886	1.014
	(3.345)	(3.374)	(3.376)	(3.362)	(3.391)	(3.413)	(3.364)	(3.396)	(3.384)
Freq. of social media use	2.001	2.008	2.071	2.128	2.132	2.139	2.054	2.052	2.107
	(1.114)	(1.119)	(1.108)	(1.121)	(1.127)	(1.123)	(1.106)	(1.111)	(1.112)
log(Total Tweets)			−0.830			−0.174			−0.321
			(0.824)			(0.790)			(0.593)
Party ID	32.987***	34.002***	33.482***	33.144***	34.142***	34.130***	33.189***	34.179***	34.107***
	(3.335)	(3.403)	(3.450)	(3.292)	(3.361)	(3.363)	(3.311)	(3.377)	(3.379)
Constant	−22.967*	−23.399*	−15.841	−22.141*	−22.565*	−21.167	−22.801*	−23.247*	−20.196
	(9.864)	(9.863)	(12.293)	(9.959)	(9.960)	(11.738)	(9.877)	(9.875)	(11.389)

*p < .05; **p < .01; ***p < .001

Supplementary Table F21: Regression models of the relationship between exposure to posts from Russian Internet Research Agency (IRA) accounts and support for expanding the Affordable Care Act

	**Expanding the ACA**								
	(1)	(2)	(3)	(4)	(5)	(6)	(7)	(8)	(9)
log(Exposure + 1)	0.159	0.179	0.375						
	(0.458)	(0.458)	(0.660)						
1(Exposure > 0)				2.246	2.287	3.905			
				(2.424)	(2.427)	(3.261)			
1(Follow > 0 IRA account)							−0.764	−0.916	−0.826
							(4.701)	(4.714)	(4.921)
Age	−0.005	−0.002	−0.010	0.004	0.007	−0.0005	−0.003	0.0002	−0.001
	(0.088)	(0.088)	(0.092)	(0.087)	(0.088)	(0.089)	(0.088)	(0.088)	(0.090)
Woman	0.246	0.272	0.297	0.328	0.349	0.361	0.175	0.191	0.186
	(2.404)	(2.400)	(2.403)	(2.393)	(2.390)	(2.389)	(2.397)	(2.394)	(2.394)
College-educated	−3.877	−3.914	−3.960	−3.883	−3.921	−4.038	−3.901	−3.941	−3.948
	(2.418)	(2.418)	(2.421)	(2.413)	(2.413)	(2.419)	(2.417)	(2.417)	(2.424)
Income	−1.249	−1.259	−1.251	−1.259	−1.270	−1.272	−1.251	−1.261	−1.261
	(1.258)	(1.259)	(1.259)	(1.254)	(1.254)	(1.254)	(1.257)	(1.257)	(1.257)
People of Color	−4.192			−4.180			−4.192		
	(3.108)			(3.111)			(3.110)		
Other/Hispanic		−1.679	−1.719		−1.660	−1.751		−1.697	−1.705
		(3.779)	(3.783)		(3.784)	(3.791)		(3.780)	(3.790)
Black		−7.731	−7.804		−7.724	−7.803		−7.705	−7.707
		(4.861)	(4.874)		(4.865)	(4.877)		(4.864)	(4.866)
Region: Northeast	3.519	3.342	3.361	3.694	3.518	3.642	3.508	3.333	3.330
	(3.584)	(3.560)	(3.557)	(3.602)	(3.577)	(3.581)	(3.590)	(3.565)	(3.566)
Region: Midwest	−0.874	−0.977	−0.902	−0.899	−1.004	−0.907	−0.901	−1.006	−0.999
	(2.953)	(2.939)	(2.963)	(2.949)	(2.936)	(2.950)	(2.955)	(2.941)	(2.954)
Region: West	−4.931	−5.407	−5.343	−4.902	−5.376	−5.188	−4.915	−5.386	−5.373
	(2.965)	(2.949)	(2.959)	(2.971)	(2.957)	(2.976)	(2.976)	(2.963)	(2.965)
Freq. of social media use	0.123	0.126	0.147	0.031	0.036	0.048	0.154	0.161	0.167
	(0.892)	(0.892)	(0.891)	(0.893)	(0.893)	(0.888)	(0.889)	(0.889)	(0.894)
log(Total Tweets)			−0.307			−0.585			−0.037
			(0.758)			(0.722)			(0.553)
Party ID	22.987***	22.390***	22.203***	23.167***	22.585***	22.555***	23.129***	22.556***	22.549***
	(3.088)	(3.144)	(3.216)	(3.094)	(3.147)	(3.151)	(3.089)	(3.142)	(3.155)
Constant	−2.286	−2.090	0.732	−3.369	−3.181	1.570	−2.226	−2.026	−1.663
	(8.137)	(8.115)	(10.955)	(8.170)	(8.146)	(10.266)	(8.146)	(8.124)	(9.955)

*p < .05; **p < .01; ***p < .001

Supplementary Table F22: Regression models of the relationship between exposure to posts from Russian Internet Research Agency (IRA) accounts and support for free trade

	**Free trade**								
	(1)	(2)	(3)	(4)	(5)	(6)	(7)	(8)	(9)
log(Exposure + 1)	0.166	0.177	0.309						
	(0.515)	(0.515)	(0.652)						
1(Exposure > 0)				0.741	0.742	1.182			
				(2.600)	(2.597)	(3.132)			
1(Follow > 0 IRA account)							5.132	5.037	5.257
							(4.935)	(4.948)	(5.110)
Age	−0.167*	−0.166*	−0.171*	−0.164*	−0.162*	−0.165*	−0.169*	−0.167*	−0.170*
	(0.082)	(0.082)	(0.084)	(0.082)	(0.082)	(0.083)	(0.081)	(0.082)	(0.083)
Woman	2.539	2.544	2.559	2.528	2.529	2.533	2.578	2.577	2.567
	(2.493)	(2.494)	(2.498)	(2.473)	(2.474)	(2.475)	(2.472)	(2.473)	(2.474)
College-educated	−0.454	−0.467	−0.491	−0.472	−0.486	−0.516	−0.403	−0.417	−0.431
	(2.562)	(2.560)	(2.553)	(2.559)	(2.558)	(2.554)	(2.563)	(2.563)	(2.559)
Income	0.274	0.262	0.268	0.266	0.254	0.254	0.244	0.233	0.232
	(1.287)	(1.287)	(1.288)	(1.285)	(1.285)	(1.285)	(1.287)	(1.287)	(1.287)
People of Color	−1.930			−1.925			−1.895		
	(3.264)			(3.264)			(3.260)		
Other/Hispanic		−0.296	−0.314		−0.311	−0.333		−0.318	−0.330
		(4.192)	(4.192)		(4.200)	(4.207)		(4.192)	(4.189)
Black		−4.259	−4.317		−4.226	−4.252		−4.144	−4.154
		(4.489)	(4.482)		(4.482)	(4.481)		(4.482)	(4.479)
Region: Northeast	−3.374	−3.488	−3.474	−3.330	−3.445	−3.408	−3.495	−3.606	−3.613
	(3.486)	(3.491)	(3.489)	(3.502)	(3.506)	(3.508)	(3.492)	(3.497)	(3.498)
Region: Midwest	−3.796	−3.863	−3.808	−3.825	−3.893	−3.867	−3.747	−3.815	−3.797
	(2.966)	(2.965)	(2.972)	(2.958)	(2.957)	(2.963)	(2.956)	(2.956)	(2.953)
Region: West	−6.129	−6.432	−6.385	−6.100	−6.398	−6.342	−6.045	−6.337	−6.302
	(3.497)	(3.484)	(3.482)	(3.499)	(3.488)	(3.481)	(3.494)	(3.482)	(3.480)
Freq. of social media use	−1.413	−1.413	−1.398	−1.424	−1.423	−1.417	−1.436	−1.433	−1.419
	(1.152)	(1.152)	(1.157)	(1.158)	(1.157)	(1.160)	(1.144)	(1.144)	(1.154)
log(Total Tweets)			−0.207			−0.159			−0.092
			(0.638)			(0.601)			(0.521)
Party ID	19.533***	19.157***	19.026***	19.675***	19.311***	19.300***	19.480***	19.128***	19.108***
	(3.331)	(3.356)	(3.388)	(3.283)	(3.308)	(3.309)	(3.297)	(3.320)	(3.321)
Constant	13.551	13.736	15.628	13.267	13.455	14.737	13.942	14.121	15.005
	(9.556)	(9.552)	(10.885)	(9.596)	(9.591)	(10.498)	(9.574)	(9.568)	(10.514)

*p < .05; **p < .01; ***p < .001

Supplementary Table F23: Regression models of the relationship between exposure to posts from Russian Internet Research Agency (IRA) accounts and opposition to tariffs with China

	**Tariffs**								
	(1)	(2)	(3)	(4)	(5)	(6)	(7)	(8)	(9)
log(Exposure + 1)	−0.019	0.015	−0.266						
	(0.641)	(0.638)	(0.817)						
1(Exposure > 0)				3.099	3.128	3.863			
				(2.788)	(2.779)	(3.400)			
1(Follow > 0 IRA account)							−8.238	−8.549	−9.555
							(7.414)	(7.256)	(7.352)
Age	−0.041	−0.034	−0.022	−0.031	−0.023	−0.027	−0.035	−0.028	−0.017
	(0.093)	(0.093)	(0.094)	(0.093)	(0.093)	(0.093)	(0.093)	(0.093)	(0.094)
Woman	−0.186	−0.160	−0.186	−0.006	0.011	0.015	−0.307	−0.296	−0.253
	(2.711)	(2.704)	(2.704)	(2.697)	(2.690)	(2.690)	(2.682)	(2.674)	(2.680)
College-educated	−3.818	−3.879	−3.822	−3.819	−3.884	−3.936	−3.913	−3.982	−3.910
	(2.769)	(2.770)	(2.767)	(2.769)	(2.770)	(2.771)	(2.761)	(2.762)	(2.757)
Income	2.010	1.962	1.956	2.008	1.959	1.955	2.043	1.995	2.003
	(1.429)	(1.424)	(1.422)	(1.427)	(1.422)	(1.422)	(1.423)	(1.417)	(1.415)
People of Color	−2.043			−2.034			−2.080		
	(3.438)			(3.435)			(3.444)		
Other/Hispanic		3.138	3.180		3.159	3.118		3.157	3.234
		(4.440)	(4.453)		(4.435)	(4.430)		(4.450)	(4.462)
Black		−9.529*	−9.411*		−9.538*	−9.587*		−9.652*	−9.590*
		(4.505)	(4.515)		(4.504)	(4.509)		(4.503)	(4.508)
Region: Northeast	−1.497	−1.930	−1.960	−1.192	−1.629	−1.567	−1.302	−1.738	−1.699
	(3.821)	(3.825)	(3.827)	(3.820)	(3.825)	(3.832)	(3.806)	(3.813)	(3.811)
Region: Midwest	1.187	1.009	0.900	1.147	0.964	1.000	1.130	0.943	0.861
	(3.310)	(3.311)	(3.312)	(3.307)	(3.309)	(3.307)	(3.303)	(3.305)	(3.303)
Region: West	−0.503	−1.443	−1.539	−0.497	−1.435	−1.346	−0.574	−1.523	−1.673
	(3.734)	(3.761)	(3.768)	(3.727)	(3.755)	(3.763)	(3.732)	(3.758)	(3.761)
Freq. of social media use	0.541	0.556	0.518	0.362	0.381	0.392	0.613	0.637	0.562
	(1.098)	(1.092)	(1.093)	(1.094)	(1.088)	(1.088)	(1.099)	(1.094)	(1.092)
log(Total Tweets)			0.440			−0.265			0.426
			(0.768)			(0.739)			(0.610)
Party ID	13.564***	12.335**	12.611**	13.640***	12.432**	12.413**	13.828***	12.619**	12.715**
	(3.895)	(3.914)	(3.974)	(3.859)	(3.877)	(3.881)	(3.870)	(3.888)	(3.896)
Constant	−9.184	−8.772	−12.772	−10.703	−10.287	−8.153	−9.728	−9.315	−13.394
	(9.980)	(9.937)	(12.025)	(10.111)	(10.072)	(11.515)	(9.954)	(9.908)	(11.535)

*p < .05; **p < .01; ***p < .001

Supplementary Table F24: Regression models of the relationship between exposure to posts from Russian Internet Research Agency (IRA) accounts and support for the use of military force

	Miltary force								
	(1)	(2)	(3)	(4)	(5)	(6)	(7)	(8)	(9)
log(Exposure + 1)	−0.565	−0.550	−1.062						
	(0.537)	(0.537)	(0.745)						
1(Exposure > 0)				−3.055	−3.029	−5.257			
				(2.446)	(2.449)	(3.162)			
1(Follow > 0 IRA account)							−6.452	−6.552	−7.060
							(7.201)	(7.177)	(7.332)
Age	−0.088	−0.085	−0.064	−0.101	−0.099	−0.088	−0.087	−0.084	−0.079
	(0.081)	(0.081)	(0.084)	(0.080)	(0.080)	(0.081)	(0.080)	(0.080)	(0.081)
Woman	0.952	0.959	0.887	0.968	0.971	0.948	1.053	1.053	1.076
	(2.295)	(2.292)	(2.294)	(2.282)	(2.280)	(2.278)	(2.291)	(2.289)	(2.289)
College-educated	−2.844	−2.868	−2.752	−2.786	−2.812	−2.643	−2.834	−2.863	−2.822
	(2.359)	(2.357)	(2.350)	(2.360)	(2.358)	(2.352)	(2.358)	(2.356)	(2.349)
Income	0.515	0.509	0.484	0.543	0.536	0.534	0.554	0.547	0.547
	(1.196)	(1.196)	(1.192)	(1.198)	(1.197)	(1.196)	(1.195)	(1.194)	(1.194)
People of Color	−2.436			−2.463			−2.499		
	(2.973)			(2.970)			(2.977)		
Other/Hispanic		−0.778	−0.680		−0.755	−0.628		−0.713	−0.671
		(3.578)	(3.565)		(3.581)	(3.567)		(3.580)	(3.567)
Black		−4.784	−4.582		−4.880	−4.776		−5.030	−5.018
		(4.580)	(4.583)		(4.563)	(4.554)		(4.590)	(4.591)
Region: Northeast	1.965	1.853	1.786	1.770	1.655	1.459	2.197	2.077	2.091
	(3.170)	(3.164)	(3.153)	(3.187)	(3.180)	(3.179)	(3.173)	(3.166)	(3.164)
Region: Midwest	7.327*	7.275*	7.077*	7.399*	7.344*	7.211*	7.341*	7.282*	7.241*
	(2.915)	(2.920)	(2.911)	(2.906)	(2.912)	(2.903)	(2.923)	(2.929)	(2.924)
Region: West	2.573	2.262	2.089	2.455	2.138	1.850	2.435	2.104	2.025
	(3.192)	(3.237)	(3.224)	(3.184)	(3.226)	(3.218)	(3.178)	(3.221)	(3.218)
Freq. of social media use	−0.345	−0.344	−0.401	−0.276	−0.273	−0.296	−0.385	−0.380	−0.412
	(0.956)	(0.956)	(0.953)	(0.962)	(0.961)	(0.959)	(0.955)	(0.954)	(0.959)
log(Total Tweets)			0.804			0.801			0.211
			(0.774)			(0.721)			(0.574)
Party ID	2.651	2.257	2.755	2.141	1.746	1.788	2.464	2.054	2.100
	(3.408)	(3.421)	(3.471)	(3.376)	(3.389)	(3.392)	(3.358)	(3.368)	(3.369)
Constant	3.385	3.506	−3.839	4.639	4.759	−1.703	2.686	2.821	0.784
	(8.421)	(8.418)	(11.381)	(8.365)	(8.361)	(10.575)	(8.482)	(8.477)	(10.157)

*p < .05; **p < .01; ***p < .001

F3 Perceived candidate polarization

Supplementary Tables F25-F33 show the complete output of issue-based and ideological positioning models presented in Figure 4B of the main text.

Supplementary Table F25: Regression models of the relationship between following a Russian Internet Research Agency (IRA) account and political ideology

	Issue-based scale (political ideology)								
	(1)	(2)	(3)	(4)	(5)	(6)	(7)	(8)	(9)
log(Exposure + 1)	0.265	0.269	0.058						
	(0.353)	(0.352)	(0.466)						
1(Exposure > 0)				1.160	1.162	0.202			
				(1.762)	(1.761)	(2.311)			
1(Follow > 0 IRA account)							4.185	4.144	3.405
							(3.116)	(3.113)	(3.260)
Age	0.003	0.004	0.013	0.009	0.010	0.015	0.002	0.003	0.011
	(0.059)	(0.059)	(0.060)	(0.059)	(0.059)	(0.059)	(0.059)	(0.059)	(0.059)
Woman	−1.737	−1.735	−1.765	−1.765	−1.764	−1.773	−1.755	−1.755	−1.724
	(1.706)	(1.706)	(1.705)	(1.704)	(1.703)	(1.703)	(1.695)	(1.695)	(1.696)
College-educated	2.055	2.038	2.085	2.025	2.008	2.081	2.074	2.058	2.116
	(1.664)	(1.665)	(1.661)	(1.662)	(1.664)	(1.666)	(1.666)	(1.668)	(1.664)
Income	−0.318	−0.321	−0.332	−0.334	−0.337	−0.336	−0.343	−0.345	−0.344
	(0.873)	(0.873)	(0.874)	(0.872)	(0.872)	(0.871)	(0.870)	(0.870)	(0.870)
People of Color	−0.301			−0.302			−0.253		
	(2.185)			(2.184)			(2.186)		
Other/Hispanic		0.429	0.471		0.404	0.468		0.417	0.465
		(2.668)	(2.665)		(2.669)	(2.663)		(2.671)	(2.664)
Black		−1.312	−1.243		−1.279	−1.234		−1.181	−1.168
		(3.372)	(3.368)		(3.372)	(3.378)		(3.382)	(3.382)
Region: Northeast	−0.034	−0.087	−0.112	0.042	−0.010	−0.100	−0.164	−0.213	−0.186
	(2.440)	(2.440)	(2.430)	(2.439)	(2.439)	(2.427)	(2.450)	(2.449)	(2.447)
Region: Midwest	−1.515	−1.555	−1.641	−1.557	−1.595	−1.652	−1.505	−1.542	−1.605
	(2.092)	(2.086)	(2.097)	(2.094)	(2.088)	(2.092)	(2.093)	(2.087)	(2.094)
Region: West	5.973**	5.830**	5.762**	6.029**	5.892**	5.771**	6.050**	5.919**	5.810**
	(2.214)	(2.221)	(2.225)	(2.214)	(2.223)	(2.225)	(2.215)	(2.224)	(2.226)
Freq. of social media use	−0.999	−0.999	−1.025	−1.021	−1.020	−1.029	−0.990	−0.989	−1.039
	(0.698)	(0.698)	(0.697)	(0.700)	(0.700)	(0.702)	(0.689)	(0.689)	(0.695)
log(Total Tweets)			0.333			0.345			0.309
			(0.497)			(0.494)			(0.389)
Party ID	1.931	1.759	1.957	2.164	2.000	2.009	2.002	1.848	1.901
	(2.358)	(2.386)	(2.377)	(2.277)	(2.311)	(2.308)	(2.292)	(2.324)	(2.311)
Constant	10.300	10.368	7.343	9.885	9.952	7.165	10.681	10.743	7.770
	(5.953)	(5.972)	(7.382)	(5.962)	(5.981)	(6.938)	(5.996)	(6.014)	(6.800)

*p < .05; **p < .01; ***p < .001

Supplementary Table F26: Regression models of the relationship between following a Russian Internet Research Agency (IRA) account and support for immigration

	Immigration								
	(1)	(2)	(3)	(4)	(5)	(6)	(7)	(8)	(9)
log(Exposure + 1)	−0.247	−0.244	−0.508						
	(0.364)	(0.363)	(0.518)						
1(Exposure > 0)				−0.475	−0.467	−1.066			
				(1.752)	(1.751)	(2.210)			
1(Follow > 0 IRA account)							−4.656	−4.680	−5.066
							(4.182)	(4.187)	(4.315)
Age	−0.062	−0.062	−0.050	−0.065	−0.065	−0.062	−0.061	−0.060	−0.056
	(0.061)	(0.062)	(0.065)	(0.062)	(0.062)	(0.063)	(0.061)	(0.062)	(0.063)
Woman	0.312	0.315	0.279	0.372	0.374	0.367	0.325	0.327	0.344
	(1.699)	(1.696)	(1.701)	(1.679)	(1.677)	(1.677)	(1.688)	(1.686)	(1.685)
College-educated	−2.612	−2.615	−2.556	−2.586	−2.591	−2.548	−2.631	−2.636	−2.606
	(1.722)	(1.723)	(1.720)	(1.722)	(1.722)	(1.720)	(1.722)	(1.722)	(1.720)
Income	−1.660	−1.660	−1.673	−1.650	−1.651	−1.651	−1.633	−1.633	−1.633
	(0.891)	(0.892)	(0.893)	(0.893)	(0.893)	(0.893)	(0.892)	(0.892)	(0.892)
People of Color	5.135*			5.121*			5.090*		
	(2.266)			(2.265)			(2.263)		
Other/Hispanic		5.471	5.512		5.491	5.521		5.500	5.526
		(3.072)	(3.077)		(3.072)	(3.072)		(3.071)	(3.071)
Black		4.644	4.734		4.582	4.607		4.492	4.488
		(2.963)	(2.972)		(2.967)	(2.973)		(2.967)	(2.966)
Region: Northeast	−0.708	−0.735	−0.758	−0.710	−0.739	−0.784	−0.578	−0.611	−0.597
	(2.597)	(2.597)	(2.590)	(2.612)	(2.612)	(2.598)	(2.600)	(2.599)	(2.605)
Region: Midwest	−3.590	−3.602	−3.694	−3.557	−3.571	−3.600	−3.609	−3.624	−3.651
	(2.094)	(2.091)	(2.086)	(2.085)	(2.083)	(2.075)	(2.098)	(2.096)	(2.090)
Region: West	−1.134	−1.197	−1.280	−1.175	−1.244	−1.314	−1.226	−1.302	−1.359
	(2.170)	(2.169)	(2.169)	(2.174)	(2.174)	(2.173)	(2.175)	(2.175)	(2.175)
Freq. of social media use	0.143	0.142	0.111	0.129	0.129	0.123	0.139	0.140	0.114
	(0.792)	(0.792)	(0.794)	(0.791)	(0.791)	(0.792)	(0.790)	(0.791)	(0.798)
log(Total Tweets)			0.416			0.216			0.162
			(0.642)			(0.577)			(0.468)
Party ID	−2.375	−2.451	−2.191	−2.567	−2.648	−2.633	−2.399	−2.489	−2.452
	(2.375)	(2.392)	(2.432)	(2.350)	(2.368)	(2.368)	(2.341)	(2.358)	(2.358)
Constant	13.992*	14.019*	10.227	14.076*	14.103*	12.352	13.604*	13.637*	12.079
	(6.817)	(6.821)	(8.860)	(6.900)	(6.904)	(8.150)	(6.839)	(6.843)	(8.026)

*p < .05; **p < .01; ***p < .001

Supplementary Table F27: Regression models of the relationship between following a Russian Internet Research Agency (IRA) account and support for banning Muslim immigrants

	Muslim ban								
	(1)	(2)	(3)	(4)	(5)	(6)	(7)	(8)	(9)
log(Exposure + 1)	−0.410	−0.426	−0.626						
	(0.403)	(0.404)	(0.544)						
1(Exposure > 0)				−1.820	−1.838	−2.459			
				(1.792)	(1.788)	(2.339)			
1(Follow > 0 IRA account)							−3.422	−3.318	−3.192
							(4.841)	(4.832)	(4.940)
Age	−0.076	−0.078	−0.070	−0.085	−0.087	−0.084	−0.076	−0.078	−0.080
	(0.064)	(0.064)	(0.065)	(0.064)	(0.064)	(0.064)	(0.064)	(0.064)	(0.064)
Woman	0.952	0.942	0.915	0.992	0.987	0.982	1.039	1.038	1.032
	(1.752)	(1.752)	(1.751)	(1.743)	(1.743)	(1.743)	(1.728)	(1.729)	(1.735)
College-educated	−1.794	−1.776	−1.735	−1.752	−1.732	−1.688	−1.784	−1.763	−1.773
	(1.812)	(1.809)	(1.803)	(1.808)	(1.804)	(1.795)	(1.811)	(1.807)	(1.807)
Income	−0.006	0.0002	−0.011	0.013	0.019	0.018	0.020	0.026	0.026
	(0.881)	(0.879)	(0.880)	(0.880)	(0.877)	(0.877)	(0.880)	(0.878)	(0.878)
People of Color	4.259			4.250			4.214		
	(2.325)			(2.323)			(2.322)		
Other/Hispanic		2.401	2.436		2.440	2.478		2.443	2.434
		(2.822)	(2.818)		(2.813)	(2.812)		(2.795)	(2.802)
Black		6.884	6.961		6.806	6.836		6.717	6.715
		(3.572)	(3.592)		(3.580)	(3.589)		(3.591)	(3.592)
Region: Northeast	−2.997	−2.861	−2.880	−3.093	−2.959	−3.006	−2.856	−2.725	−2.730
	(2.583)	(2.586)	(2.582)	(2.580)	(2.583)	(2.575)	(2.588)	(2.591)	(2.593)
Region: Midwest	−4.643*	−4.582*	−4.658*	−4.584*	−4.522*	−4.556*	−4.629*	−4.568*	−4.558*
	(2.124)	(2.125)	(2.128)	(2.115)	(2.117)	(2.116)	(2.125)	(2.127)	(2.124)
Region: West	−1.975	−1.627	−1.689	−2.056	−1.720	−1.796	−2.072	−1.742	−1.723
	(2.377)	(2.368)	(2.360)	(2.378)	(2.369)	(2.368)	(2.380)	(2.371)	(2.366)
Freq. of social media use	0.618	0.616	0.593	0.649	0.646	0.639	0.579	0.574	0.583
	(0.964)	(0.964)	(0.963)	(0.960)	(0.960)	(0.959)	(0.964)	(0.964)	(0.968)
log(Total Tweets)			0.314			0.224			−0.053
			(0.511)			(0.495)			(0.384)
Party ID	−5.650*	−5.221*	−5.030*	−5.998*	−5.591*	−5.579*	−5.839*	−5.443*	−5.454*
	(2.385)	(2.417)	(2.450)	(2.408)	(2.443)	(2.444)	(2.405)	(2.440)	(2.450)
Constant	9.448	9.318	6.458	10.137	10.010	8.207	9.011	8.882	9.390
	(8.248)	(8.248)	(9.171)	(8.356)	(8.358)	(8.906)	(8.263)	(8.263)	(8.951)

*p < .05; **p < .01; ***p < .001

Supplementary Table F28: Regression models of the relationship between following a Russian Internet Research Agency (IRA) account and support for building a wall on the border with Mexico

	Building a wall								
	(1)	(2)	(3)	(4)	(5)	(6)	(7)	(8)	(9)
log(Exposure + 1)	−0.855*	−0.878*	−0.859						
	(0.371)	(0.368)	(0.519)						
1(Exposure > 0)				−3.321	−3.355	−2.669			
				(1.794)	(1.789)	(2.294)			
1(Follow > 0 IRA account)							−7.025	−6.884	−5.665
							(4.189)	(4.119)	(4.234)
Age	0.013	0.009	0.008	−0.003	−0.007	−0.011	0.013	0.009	−0.004
	(0.064)	(0.064)	(0.066)	(0.064)	(0.064)	(0.064)	(0.064)	(0.064)	(0.065)
Woman	1.012	1.005	1.008	1.126	1.127	1.131	1.214	1.219	1.156
	(1.719)	(1.717)	(1.719)	(1.706)	(1.705)	(1.704)	(1.706)	(1.704)	(1.708)
College-educated	−2.236	−2.205	−2.209	−2.147	−2.115	−2.164	−2.220	−2.187	−2.277
	(1.757)	(1.751)	(1.755)	(1.758)	(1.753)	(1.757)	(1.757)	(1.752)	(1.758)
Income	−2.421**	−2.414**	−2.413**	−2.382**	−2.374**	−2.373**	−2.371**	−2.363**	−2.361**
	(0.855)	(0.851)	(0.852)	(0.857)	(0.853)	(0.853)	(0.856)	(0.852)	(0.851)
People of Color	3.459			3.435			3.369		
	(2.390)			(2.398)			(2.404)		
Other/Hispanic		0.855	0.851		0.927	0.884		0.947	0.847
		(2.795)	(2.799)		(2.814)	(2.823)		(2.801)	(2.810)
Black		7.164	7.156		7.002	6.968		6.816	6.794
		(3.869)	(3.889)		(3.875)	(3.883)		(3.905)	(3.895)
Region: Northeast	3.294	3.479	3.480	3.146	3.325	3.376	3.584	3.758	3.710
	(2.664)	(2.657)	(2.653)	(2.676)	(2.670)	(2.662)	(2.670)	(2.665)	(2.670)
Region: Midwest	−0.681	−0.600	−0.593	−0.557	−0.476	−0.441	−0.650	−0.570	−0.477
	(2.097)	(2.087)	(2.096)	(2.091)	(2.081)	(2.082)	(2.097)	(2.087)	(2.087)
Region: West	0.415	0.902	0.909	0.266	0.731	0.814	0.228	0.679	0.858
	(2.239)	(2.205)	(2.202)	(2.239)	(2.208)	(2.209)	(2.248)	(2.215)	(2.207)
Freq. of social media use	−0.224	−0.226	−0.224	−0.191	−0.194	−0.187	−0.313	−0.319	−0.238
	(0.771)	(0.770)	(0.775)	(0.773)	(0.771)	(0.774)	(0.772)	(0.770)	(0.780)
log(Total Tweets)			−0.030			−0.248			−0.508
			(0.619)			(0.566)			(0.452)
Party ID	−1.737	−1.125	−1.144	−2.435	−1.862	−1.878	−2.118	−1.568	−1.679
	(2.442)	(2.456)	(2.502)	(2.373)	(2.391)	(2.392)	(2.395)	(2.410)	(2.408)
Constant	13.271	13.092	13.368	14.472*	14.304*	16.301	12.403	12.229	17.115*
	(7.153)	(7.139)	(8.926)	(7.191)	(7.179)	(8.390)	(7.196)	(7.180)	(8.257)

*p < .05; **p < .01; ***p < .001

Supplementary Table F29: Regression models of the relationship between following a Russian Internet Research Agency (IRA) account and support for Obamacare

	Obamacare								
	(1)	(2)	(3)	(4)	(5)	(6)	(7)	(8)	(9)
log(Exposure + 1)	−0.281	−0.238	−0.214						
	(0.398)	(0.396)	(0.557)						
1(Exposure > 0)				−0.686	−0.612	−0.181			
				(2.056)	(2.044)	(2.659)			
1(Follow > 0 IRA account)							−0.171	−0.461	−0.040
							(4.209)	(4.164)	(4.304)
Age	0.012	0.017	0.016	0.008	0.014	0.012	0.010	0.016	0.012
	(0.066)	(0.066)	(0.067)	(0.066)	(0.067)	(0.066)	(0.066)	(0.066)	(0.066)
Woman	−2.197	−2.164	−2.161	−2.138	−2.116	−2.113	−2.101	−2.088	−2.107
	(1.932)	(1.925)	(1.925)	(1.927)	(1.920)	(1.920)	(1.923)	(1.916)	(1.918)
College-educated	0.669	0.592	0.587	0.694	0.612	0.583	0.692	0.608	0.578
	(1.966)	(1.957)	(1.955)	(1.968)	(1.959)	(1.957)	(1.969)	(1.960)	(1.956)
Income	2.140*	2.129*	2.130*	2.153*	2.140*	2.140*	2.150*	2.138*	2.139*
	(1.021)	(1.013)	(1.012)	(1.018)	(1.011)	(1.011)	(1.020)	(1.012)	(1.011)
People of Color	−0.884			−0.892			−0.894		
	(2.524)			(2.527)			(2.526)		
Other/Hispanic		3.796	3.791		3.820	3.790		3.827	3.787
		(3.367)	(3.366)		(3.370)	(3.366)		(3.370)	(3.366)
Black		−7.325*	−7.334*		−7.374*	−7.397*		−7.394*	−7.403*
		(3.108)	(3.118)		(3.115)	(3.117)		(3.118)	(3.117)
Region: Northeast	0.962	0.639	0.641	0.944	0.619	0.653	1.014	0.687	0.671
	(2.661)	(2.650)	(2.650)	(2.676)	(2.666)	(2.675)	(2.670)	(2.659)	(2.658)
Region: Midwest	−1.835	−2.000	−1.991	−1.796	−1.968	−1.943	−1.798	−1.973	−1.940
	(2.538)	(2.530)	(2.548)	(2.537)	(2.529)	(2.540)	(2.537)	(2.528)	(2.533)
Region: West	1.691	0.773	0.781	1.628	0.714	0.770	1.622	0.706	0.778
	(2.591)	(2.597)	(2.598)	(2.586)	(2.593)	(2.601)	(2.584)	(2.591)	(2.596)
Freq. of social media use	0.308	0.300	0.302	0.295	0.290	0.296	0.258	0.260	0.291
	(0.839)	(0.838)	(0.842)	(0.846)	(0.845)	(0.844)	(0.833)	(0.832)	(0.841)
log(Total Tweets)			−0.036			−0.156			−0.180
			(0.688)			(0.646)			(0.513)
Party ID	2.203	1.111	1.088	1.983	0.916	0.906	2.015	0.951	0.910
	(2.860)	(2.898)	(2.913)	(2.822)	(2.862)	(2.863)	(2.837)	(2.875)	(2.873)
Constant	9.889	10.353	10.685	10.090	10.541	11.794	9.737	10.215	11.927
	(7.180)	(7.172)	(9.149)	(7.282)	(7.275)	(8.492)	(7.188)	(7.181)	(8.376)

*p < .05; **p < .01; ***p < .001

Supplementary Table F30: Regression models of the relationship between following a Russian Internet Research Agency (IRA) account and support for expanding the Affordable Care Act

	Expanding the ACA								
	(1)	(2)	(3)	(4)	(5)	(6)	(7)	(8)	(9)
log(Exposure + 1)	−0.672	−0.663	−0.458						
	(0.383)	(0.382)	(0.530)						
1(Exposure > 0)				−2.495	−2.477	−1.160			
				(1.876)	(1.876)	(2.391)			
1(Follow > 0 IRA account)							−6.072	−6.147	−4.842
							(4.207)	(4.204)	(4.343)
Age	0.039	0.040	0.032	0.026	0.028	0.021	0.038	0.040	0.027
	(0.062)	(0.063)	(0.065)	(0.063)	(0.063)	(0.064)	(0.062)	(0.063)	(0.064)
Woman	−3.831*	−3.820*	−3.793*	−3.740*	−3.731*	−3.721*	−3.688*	−3.680*	−3.741*
	(1.850)	(1.847)	(1.848)	(1.853)	(1.851)	(1.850)	(1.851)	(1.850)	(1.847)
College-educated	−0.095	−0.111	−0.159	−0.038	−0.056	−0.151	−0.084	−0.104	−0.208
	(1.806)	(1.810)	(1.813)	(1.808)	(1.812)	(1.813)	(1.809)	(1.813)	(1.814)
Income	−0.071	−0.075	−0.067	−0.042	−0.047	−0.049	−0.023	−0.028	−0.032
	(0.966)	(0.967)	(0.967)	(0.965)	(0.967)	(0.967)	(0.964)	(0.966)	(0.967)
People of Color	0.489			0.460			0.430		
	(2.419)			(2.420)			(2.416)		
Other/Hispanic		1.575	1.533		1.607	1.534		1.655	1.544
		(3.229)	(3.235)		(3.230)	(3.234)		(3.216)	(3.231)
Black		−1.039	−1.117		−1.154	−1.219		−1.295	−1.324
		(3.266)	(3.278)		(3.274)	(3.278)		(3.283)	(3.276)
Region: Northeast	−1.581	−1.657	−1.637	−1.697	−1.777	−1.677	−1.355	−1.441	−1.484
	(2.659)	(2.653)	(2.648)	(2.679)	(2.673)	(2.664)	(2.671)	(2.664)	(2.664)
Region: Midwest	−2.738	−2.782	−2.703	−2.654	−2.702	−2.623	−2.726	−2.778	−2.667
	(2.239)	(2.239)	(2.241)	(2.242)	(2.241)	(2.241)	(2.242)	(2.241)	(2.238)
Region: West	1.773	1.568	1.635	1.664	1.449	1.602	1.601	1.370	1.563
	(2.415)	(2.405)	(2.407)	(2.412)	(2.404)	(2.411)	(2.415)	(2.408)	(2.403)
Freq. of social media use	0.572	0.573	0.594	0.596	0.598	0.608	0.514	0.518	0.598
	(0.686)	(0.683)	(0.686)	(0.694)	(0.691)	(0.690)	(0.686)	(0.683)	(0.687)
log(Total Tweets)			−0.323			−0.476			−0.547
			(0.586)			(0.541)			(0.436)
Party ID	5.071*	4.813	4.617	4.508	4.243	4.218	4.779	4.497	4.390
	(2.543)	(2.576)	(2.611)	(2.519)	(2.553)	(2.553)	(2.520)	(2.554)	(2.554)
Constant	10.481	10.565	13.529	11.373	11.458	15.325*	9.749	9.847	15.158*
	(6.198)	(6.202)	(8.228)	(6.298)	(6.302)	(7.642)	(6.198)	(6.203)	(7.547)

*p < .05; **p < .01; ***p < .001

Supplementary Table F31: Regression models of the relationship between following a Russian Internet Research Agency (IRA) account and support for free trade

	Free trade								
	(1)	(2)	(3)	(4)	(5)	(6)	(7)	(8)	(9)
log(Exposure + 1)	−0.617	−0.627	−1.198*						
	(0.420)	(0.419)	(0.543)						
1(Exposure > 0)				0.723	0.720	0.746			
				(2.093)	(2.090)	(2.648)			
1(Follow > 0 IRA account)							−10.737*	−10.657*	−11.357*
							(4.350)	(4.326)	(4.417)
Age	0.041	0.040	0.064	0.040	0.039	0.039	0.043	0.042	0.049
	(0.067)	(0.067)	(0.068)	(0.067)	(0.067)	(0.068)	(0.067)	(0.067)	(0.068)
Woman	−2.995	−3.004	−3.077	−2.731	−2.737	−2.737	−2.980	−2.984	−2.955
	(1.950)	(1.950)	(1.952)	(1.945)	(1.945)	(1.945)	(1.937)	(1.937)	(1.937)
College-educated	−1.066	−1.056	−0.951	−0.999	−0.989	−0.990	−1.145	−1.134	−1.090
	(2.092)	(2.092)	(2.089)	(2.093)	(2.093)	(2.091)	(2.090)	(2.090)	(2.089)
Income	−1.668	−1.656	−1.680	−1.647	−1.635	−1.635	−1.595	−1.585	−1.583
	(1.055)	(1.054)	(1.054)	(1.057)	(1.056)	(1.056)	(1.052)	(1.051)	(1.051)
People of Color	3.245			3.213			3.161		
	(2.628)			(2.630)			(2.627)		
Other/Hispanic		1.768	1.852		1.806	1.804		1.829	1.869
		(3.478)	(3.456)		(3.476)	(3.481)		(3.467)	(3.460)
Black		5.340	5.563		5.209	5.208		5.053	5.078
		(3.433)	(3.455)		(3.442)	(3.446)		(3.457)	(3.466)
Region: Northeast	−1.487	−1.385	−1.446	−1.305	−1.206	−1.204	−1.184	−1.092	−1.068
	(2.810)	(2.800)	(2.792)	(2.826)	(2.816)	(2.811)	(2.800)	(2.790)	(2.792)
Region: Midwest	−1.812	−1.761	−2.013	−1.697	−1.646	−1.644	−1.865	−1.816	−1.875
	(2.417)	(2.418)	(2.424)	(2.409)	(2.409)	(2.409)	(2.414)	(2.415)	(2.414)
Region: West	1.902	2.183	1.986	1.799	2.064	2.068	1.680	1.933	1.825
	(2.703)	(2.691)	(2.675)	(2.704)	(2.693)	(2.684)	(2.697)	(2.685)	(2.677)
Freq. of social media use	−0.650	−0.650	−0.716	−0.795	−0.797	−0.796	−0.649	−0.651	−0.698
	(0.968)	(0.970)	(0.968)	(0.965)	(0.966)	(0.968)	(0.953)	(0.954)	(0.961)
log(Total Tweets)			0.898			−0.009			0.292
			(0.543)			(0.531)			(0.428)
Party ID	−0.243	0.097	0.661	−0.687	−0.370	−0.370	−0.335	−0.037	0.026
	(2.769)	(2.783)	(2.783)	(2.736)	(2.747)	(2.746)	(2.737)	(2.745)	(2.740)
Constant	18.101*	17.950*	9.774	17.367*	17.220*	17.294	17.122*	16.985*	14.181
	(8.349)	(8.364)	(9.402)	(8.342)	(8.357)	(9.037)	(8.328)	(8.339)	(8.917)

*p < .05; **p < .01; ***p < .001

Supplementary Table F32: Regression models of the relationship between following a Russian Internet Research Agency (IRA) account and opposition to tariffs with China

	**Tariffs**								
	(1)	(2)	(3)	(4)	(5)	(6)	(7)	(8)	(9)
log(Exposure + 1)	−1.049*	−1.057*	−1.374*						
	(0.465)	(0.464)	(0.603)						
1(Exposure > 0)				−2.971	−2.977	−2.872			
				(2.146)	(2.144)	(2.698)			
1(Follow > 0 IRA account)							−7.685	−7.623	−6.893
							(5.295)	(5.284)	(5.363)
Age	0.018	0.016	0.029	0.002	−0.0001	−0.001	0.017	0.015	0.007
	(0.072)	(0.073)	(0.074)	(0.072)	(0.072)	(0.073)	(0.072)	(0.072)	(0.073)
Woman	1.550	1.544	1.514	1.711	1.707	1.708	1.759	1.756	1.725
	(2.080)	(2.079)	(2.077)	(2.076)	(2.074)	(2.074)	(2.061)	(2.060)	(2.066)
College-educated	3.152	3.166	3.231	3.265	3.279	3.271	3.172	3.186	3.134
	(2.139)	(2.137)	(2.136)	(2.141)	(2.141)	(2.142)	(2.136)	(2.135)	(2.139)
Income	−0.889	−0.878	−0.885	−0.845	−0.835	−0.835	−0.818	−0.808	−0.814
	(1.109)	(1.108)	(1.109)	(1.116)	(1.115)	(1.114)	(1.114)	(1.113)	(1.110)
People of Color	5.777*			5.784*			5.758*		
	(2.637)			(2.644)			(2.655)		
Other/Hispanic		4.565	4.613		4.674	4.668		4.714	4.658
		(3.195)	(3.190)		(3.216)	(3.222)		(3.211)	(3.216)
Black		7.528	7.662		7.388	7.381		7.268	7.223
		(3.904)	(3.925)		(3.905)	(3.912)		(3.950)	(3.940)
Region: Northeast	−3.775	−3.674	−3.708	−3.879	−3.786	−3.777	−3.411	−3.324	−3.352
	(2.861)	(2.861)	(2.859)	(2.889)	(2.890)	(2.893)	(2.855)	(2.855)	(2.856)
Region: Midwest	−1.329	−1.287	−1.410	−1.164	−1.125	−1.120	−1.261	−1.224	−1.164
	(2.589)	(2.585)	(2.589)	(2.586)	(2.581)	(2.579)	(2.583)	(2.579)	(2.577)
Region: West	−2.075	−1.855	−1.963	−2.245	−2.045	−2.032	−2.301	−2.112	−2.003
	(2.892)	(2.879)	(2.878)	(2.892)	(2.882)	(2.879)	(2.902)	(2.893)	(2.895)
Freq. of social media use	0.036	0.033	−0.010	0.021	0.017	0.019	−0.077	−0.082	−0.027
	(1.001)	(1.002)	(1.002)	(0.997)	(0.997)	(0.999)	(0.992)	(0.993)	(1.001)
log(Total Tweets)			0.498			−0.038			−0.309
			(0.623)			(0.602)			(0.485)
Party ID	3.699	3.987	4.299	2.855	3.114	3.111	3.199	3.440	3.370
	(3.019)	(3.029)	(3.083)	(3.000)	(3.015)	(3.017)	(3.021)	(3.029)	(3.033)
Constant	3.317	3.221	−1.307	4.218	4.129	4.434	2.272	2.189	5.146
	(8.476)	(8.473)	(9.968)	(8.543)	(8.541)	(9.587)	(8.489)	(8.484)	(9.440)

*p < .05; **p < .01; ***p < .001

Supplementary Table F33: Regression models of the relationship between following a Russian Internet Research Agency (IRA) account and support for the use of military force

	**Miltary force**								
	(1)	(2)	(3)	(4)	(5)	(6)	(7)	(8)	(9)
log(Exposure + 1)	0.096	0.097	0.087						
	(0.440)	(0.439)	(0.600)						
1(Exposure > 0)				1.794	1.795	2.560			
				(1.915)	(1.914)	(2.350)			
1(Follow > 0 IRA account)							−4.835	−4.840	−5.245
							(5.370)	(5.382)	(5.507)
Age	0.035	0.035	0.035	0.042	0.042	0.038	0.038	0.038	0.043
	(0.067)	(0.067)	(0.068)	(0.067)	(0.067)	(0.067)	(0.067)	(0.067)	(0.067)
Woman	1.211	1.211	1.210	1.289	1.289	1.297	1.093	1.093	1.111
	(1.859)	(1.857)	(1.860)	(1.849)	(1.848)	(1.847)	(1.847)	(1.847)	(1.848)
College-educated	−1.870	−1.870	−1.868	−1.876	−1.877	−1.935	−1.924	−1.925	−1.892
	(1.905)	(1.907)	(1.902)	(1.901)	(1.903)	(1.900)	(1.899)	(1.900)	(1.898)
Income	−2.185*	−2.186*	−2.186*	−2.192*	−2.192*	−2.192*	−2.178*	−2.178*	−2.178*
	(1.016)	(1.017)	(1.017)	(1.014)	(1.015)	(1.015)	(1.010)	(1.011)	(1.011)
People of Color	0.380			0.388			0.349		
	(2.472)			(2.467)			(2.478)		
Other/Hispanic		0.445	0.447		0.460	0.416		0.433	0.466
		(3.090)	(3.081)		(3.087)	(3.076)		(3.089)	(3.077)
Black		0.287	0.290		0.286	0.250		0.231	0.240
		(3.739)	(3.751)		(3.733)	(3.734)		(3.767)	(3.769)
Region: Northeast	−2.868	−2.872	−2.874	−2.714	−2.719	−2.652	−2.780	−2.786	−2.775
	(2.779)	(2.773)	(2.775)	(2.799)	(2.791)	(2.806)	(2.781)	(2.773)	(2.774)
Region: Midwest	−2.914	−2.916	−2.920	−2.927	−2.929	−2.884	−2.969	−2.971	−3.003
	(2.279)	(2.281)	(2.288)	(2.273)	(2.275)	(2.280)	(2.281)	(2.283)	(2.281)
Region: West	−0.570	−0.583	−0.586	−0.550	−0.563	−0.464	−0.567	−0.582	−0.645
	(2.538)	(2.560)	(2.560)	(2.537)	(2.559)	(2.571)	(2.532)	(2.554)	(2.552)
Freq. of social media use	−0.389	−0.389	−0.390	−0.467	−0.467	−0.459	−0.335	−0.335	−0.361
	(0.735)	(0.735)	(0.738)	(0.738)	(0.738)	(0.740)	(0.729)	(0.729)	(0.737)
log(Total Tweets)			0.015			−0.275			0.168
			(0.674)			(0.612)			(0.509)
Party ID	−5.607*	−5.622*	−5.613*	−5.475*	−5.492*	−5.506*	−5.375*	−5.394*	−5.357*
	(2.725)	(2.754)	(2.784)	(2.699)	(2.731)	(2.735)	(2.701)	(2.729)	(2.728)
Constant	12.700*	12.705*	12.570	11.806	11.811	14.032	12.524	12.530	10.904
	(6.402)	(6.412)	(8.656)	(6.445)	(6.455)	(8.030)	(6.405)	(6.414)	(7.849)

*p < .05; **p < .01; ***p < .001

F4 Alternative coding of race

For ease of comparison in Supplementary Figure F11 we present a subset of the models from Supplementary Tables F16-F24 and Supplementary Tables F25-F33 above, which is analogous to Figure 4 of the main text, but has an alternative coding of control variable race. Here, rather than treating all respondents of color similarly, we break them further down into Black and Hispanic/Other groups. Consistent with the results in the main manuscript, we find no evidence of a relationship between exposure to Internet Research Agency accounts and changes in issue-based or ideological positioning.

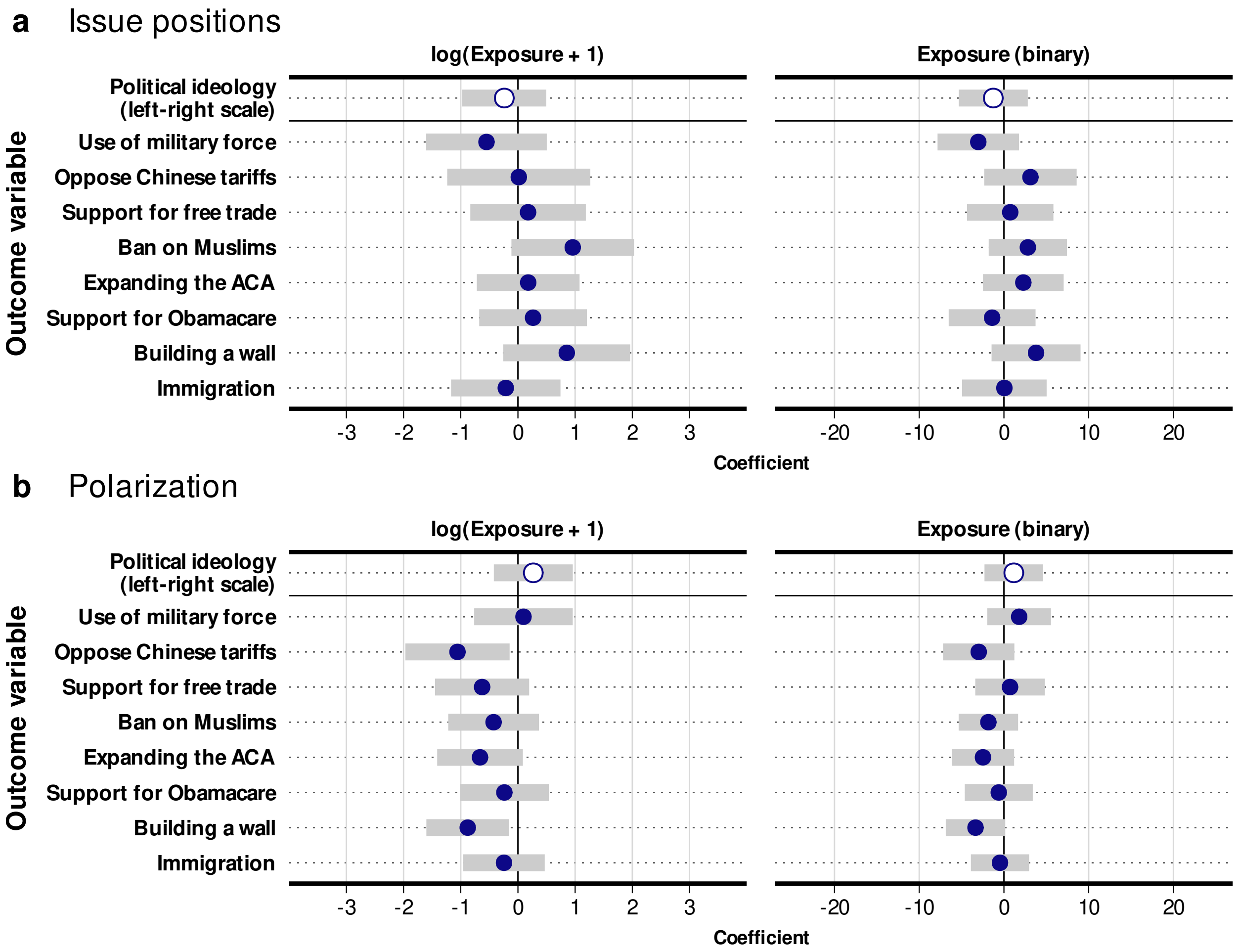

Supplementary Figure F11: Regression results of exposure to Russian Internet Research Agency accounts and changes in issue-based and ideological positioning with alternative race break-down. Error bars represent 95% confidence intervals around point estimates of the regression coefficients. $n = 1,496$ survey respondents

F5 Alternative measure of overall Twitter activity

As another robustness check, rather than relying on self-reported measure of social media activity, that might make respondents less likely to be exposed to tweets from foreign influence campaigns, we control for the total number of tweets posted by their friends. In constructing this measure we rely on our own collected data to estimate the number of potential exposures to other tweets (non-Internet Research Agency) in respondents' timelines. These models are also presented in Supplementary Tables F16-F24 and Supplementary Tables F25-F33 above, but for ease of comparison to Figure 4 of the main manuscript in Supplementary Figure F12 we present a subset of them that use log(Total Tweets) as one of control variables. Consistent with the results in the main manuscript, we find no evidence of a relationship between exposure to Internet Research Agency accounts and changes in issue-based or ideological positioning.

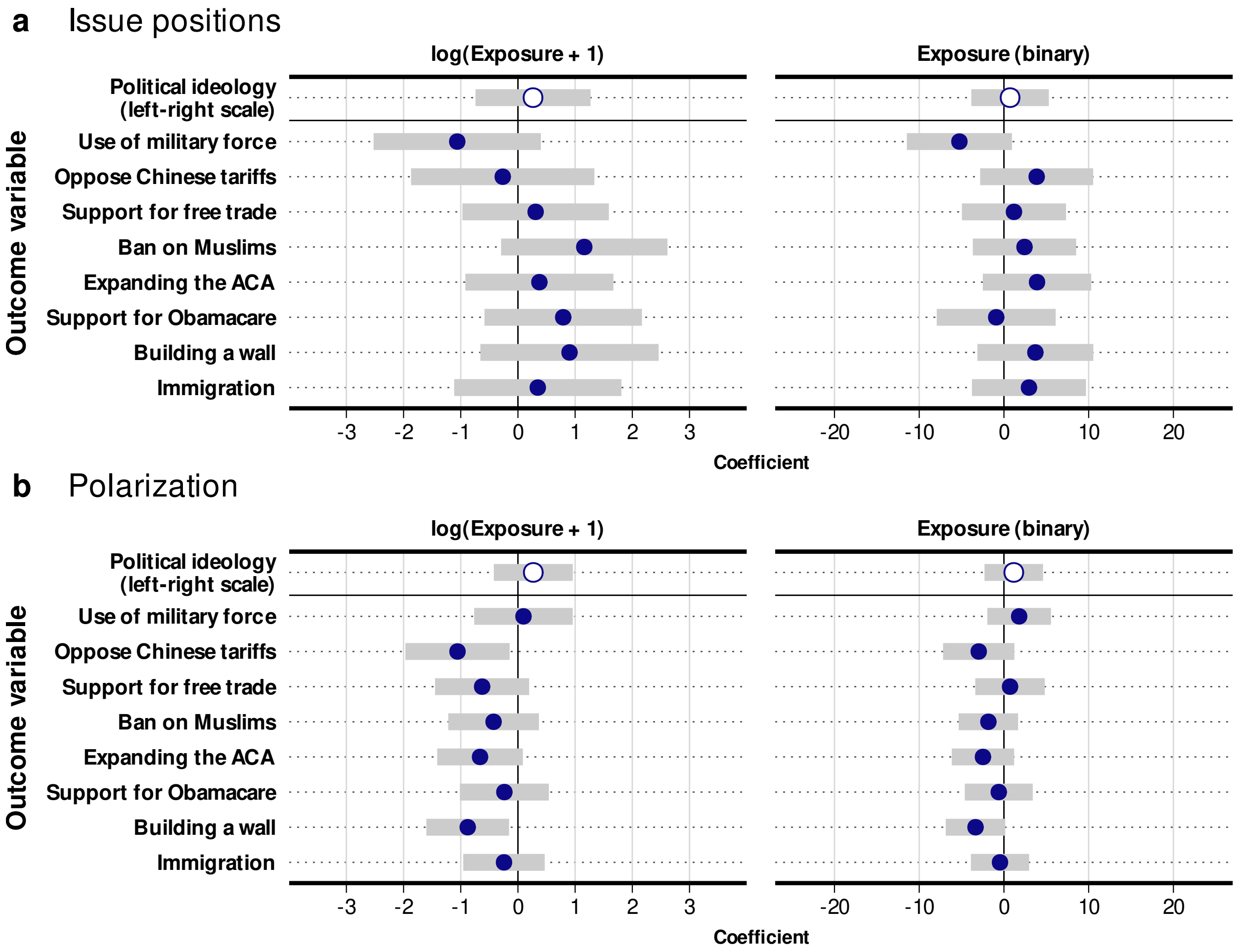

Supplementary Figure F12: Regression results of exposure to Russian Internet Research Agency accounts and changes in issue-based and ideological positioning with alternative measure of overall potential exposures on Twitter. Error bars represent 95% confidence intervals around point estimates of the regression coefficients. $n = 1,496$ survey respondents

G Adjustment for multiple hypothesis testing

Supplementary Figure G13 presents the results of the OLS models showing the relationship between exposure on issue positions and polarization with Bonferroni corrections applied to address multiple comparisons. Supplementary Figure G14 shows corresponding results of the OLS models from Figure 5 of the main manuscript after adjusting for multiple comparisons with Bonferroni correction.

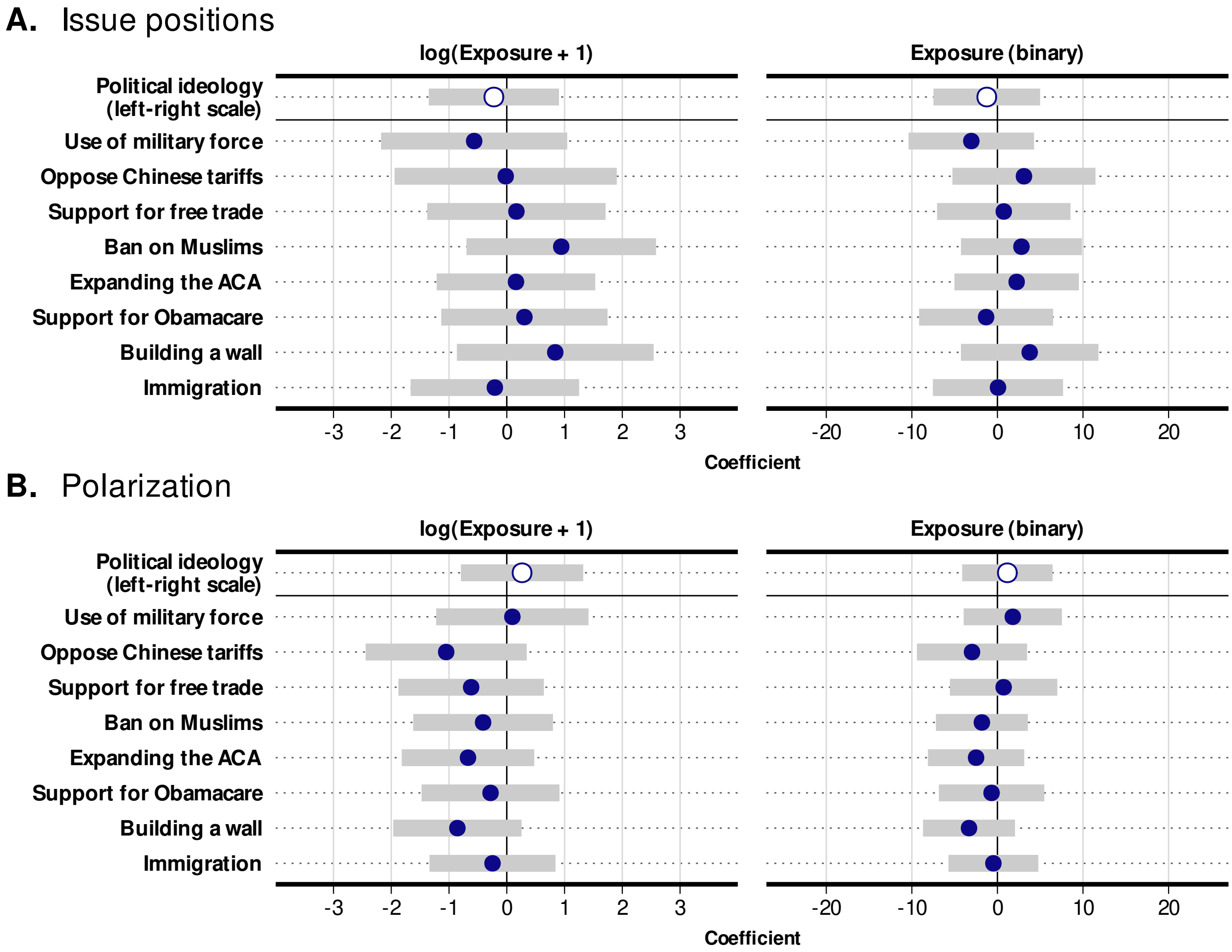

Supplementary Figure G13: Regression results of exposure to Russian Internet Research Agency accounts and changes in issue-based and ideological positionin. Point estimates from OLS models with 95% CIs adjusted for multiple comparisons using Bonferroni correction. $n = 1,496$ survey respondents

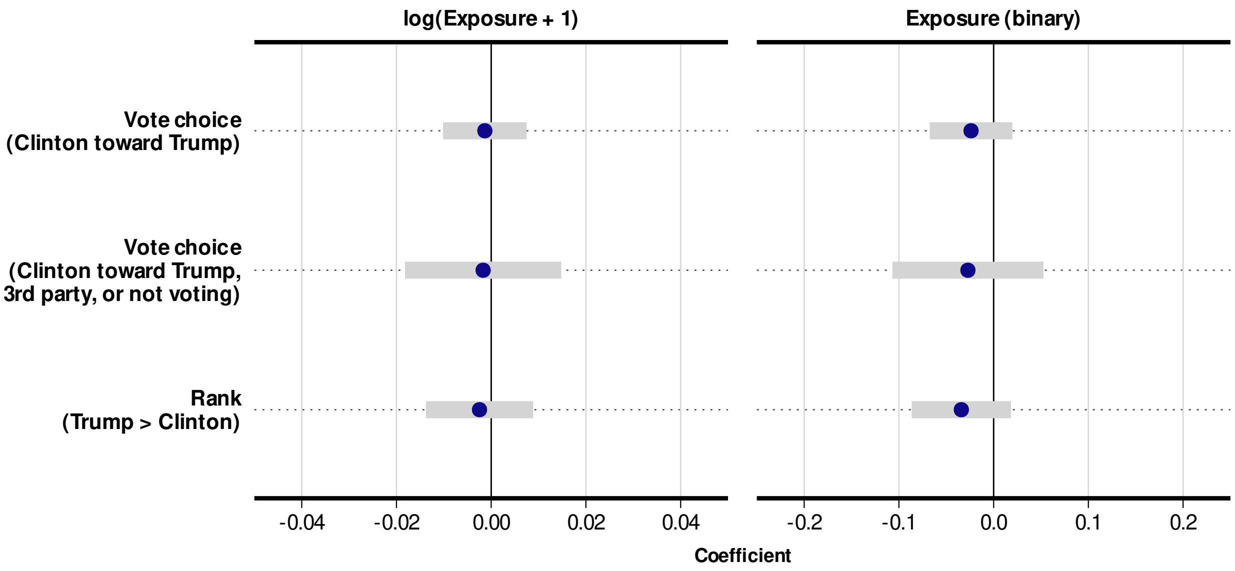

Supplementary Figure G14: Regression results of the relationship between exposure to posts from Russian Internet Research Agency accounts and voting behavior. Point estimates from OLS models with 95% CIs adjusted for multiple comparisons using Bonferroni correction. $n = 1,496$ survey respondents

Supplementary References

[1] P. D. Allison. Change scores as dependent variables in regression analysis. *Sociological Methodology*, 20:93–114, 1990.

[2] J. Cohen. *Statistical Power Analysis for the Behavioral Sciences.* Lawrence Erlbaum Associates, New York, NY, 1969.

[3] N. Grinberg, K. Joseph, L. Friedland, B. Swire-Thompson, and D. Lazer. Fake news on Twitter during the 2016 U.S. presidential election. *Science*, 363(6425):374–378, 2019. ISSN 10959203. doi: 10.1126/science.aau2706.

[4] E. Hartman and F. D. Hidalgo. An equivalence approach to balance and placebo tests. *American Journal of Political Science*, 62(4):1000–1013, 2018.

[5] D. E. Ho, K. Imai, G. King, and E. A. Stuart. Matching as nonparametric preprocessing for reducing model dependence in parametric causal inference. *Political Analysis*, 15(3): 199–236, 2007.

[6] G. W. Imbens and D. B. Rubin. *Causal Inference for Statistics, Social, and Biomedical Sciences: An Introduction.* Cambridge University Press, New York, NY, 2015.

[7] D. Lakens. Equivalence tests: A practical primer for t tests, correlations, and meta-analyses. *Social Psychological and Personality Science*, 8(4):355–362, 2017.

[8] C. Rainey. Arguing for a negligible effect. *American Journal of Political Science*, 58(4): 1083–1091, 2014.
