# Supplementary material for: Exposure to the Russian Internet Research Agency foreign influence campaign on Twitter in the 2016 US election and its relationship to attitudes and voting behavior
Source: Nat Commun. 2023 Jan 9;14:62. doi: 10.1038/s41467-022-35576-9 (PMC9829855; doi:10.1038/s41467-022-35576-9)
Supplement: Supplementary file 3 — Reporting Summary [file 41467_2022_35576_MOESM3_ESM.pdf]

nature portfolio

Corresponding author(s): Gregory Eady

Last updated by author(s): Sep 15, 2022

Reporting Summary

Nature Portfolio wishes to improve the reproducibility of the work that we publish. This form provides structure for consistency and transparency in reporting. For further information on Nature Portfolio policies, see our Editorial Policies and the Editorial Policy Checklist.

Statistics

For all statistical analyses, confirm that the following items are present in the figure legend, table legend, main text, or Methods section.

n/a	Confirmed	
☐	☒	The exact sample size (*n*) for each experimental group/condition, given as a discrete number and unit of measurement
☐	☒	A statement on whether measurements were taken from distinct samples or whether the same sample was measured repeatedly
☐	☒	The statistical test(s) used AND whether they are one- or two-sided<br>*Only common tests should be described solely by name; describe more complex techniques in the Methods section.*
☐	☒	A description of all covariates tested
☐	☒	A description of any assumptions or corrections, such as tests of normality and adjustment for multiple comparisons
☐	☒	A full description of the statistical parameters including central tendency (e.g. means) or other basic estimates (e.g. regression coefficient) AND variation (e.g. standard deviation) or associated estimates of uncertainty (e.g. confidence intervals)
☐	☒	For null hypothesis testing, the test statistic (e.g. *F*, *t*, *r*) with confidence intervals, effect sizes, degrees of freedom and *P* value noted<br>*Give P values as exact values whenever suitable.*
☒	☐	For Bayesian analysis, information on the choice of priors and Markov chain Monte Carlo settings
☒	☐	For hierarchical and complex designs, identification of the appropriate level for tests and full reporting of outcomes
☐	☒	Estimates of effect sizes (e.g. Cohen's *d*, Pearson's *r*), indicating how they were calculated

Our web collection on statistics for biologists contains articles on many of the points above.

Software and code

Policy information about availability of computer code

Data collection	Survey data was collected by a survey firm YouGov using their proprietary survey platform. Twitter Elections Integrity data was downloaded from the official Twitter repository. Information on friends and followers of survey respondents was collected using an original Python script.
Data analysis	Analyses were with R (4.1.3), Python (3.7), cowplot (1.1.1), dplyr (1.0.7), estimatr (0.30.2), ggplot2 (3.3.6), haven (2.4.3), knitr (1.37), lmtest (0.9.40), MASS (7.3.55), mvtnorm (1.1.2), readr (2.1.1), stringi (1.7.6), stringr (1.4.0), tibble (3.1.7), tidyr (1.1.4), and quanteda (3.1.0).

For manuscripts utilizing custom algorithms or software that are central to the research but not yet described in published literature, software must be made available to editors and reviewers. We strongly encourage code deposition in a community repository (e.g. GitHub). See the Nature Portfolio guidelines for submitting code & software for further information.

Data

Policy information about availability of data

All manuscripts must include a data availability statement. This statement should provide the following information, where applicable:

- Accession codes, unique identifiers, or web links for publicly available datasets
- A description of any restrictions on data availability
- For clinical datasets or third party data, please ensure that the statement adheres to our policy

The authors have deposited all replication materials, including minimal datasets required to replicate the methods used in this paper in a GitHub repository located at: https://github.com/tpaskhalis/ncomms_russia_us_2016. All other relevant data are available upon reasonable request to the authors. Full data are not publicly

available due to terms of data license agreement signed with Twitter, Inc. and survey data containing information that could compromise research participant privacy. Source data are provided with this paper.

Human research participants

Policy information about studies involving human research participants and Sex and Gender in Research.

Reporting on sex and gender	Gender is not a key variable in any of the analyses. As a control in one model, the gender of a survey respondent refers to their response to a question that asks them to state their gender.
Population characteristics	U.S. adult population registered on Twitter (n = 1496)). Male (44%), female (56%), age 18-29 (13%), age 30-49 (40%), age 50-64 (33%), age 65 and older (14%), college graduate (35%), non-college graduate (65%)
Recruitment	Participants were recruited via email by the Internet survey firm YouGov to members of their proprietary panel. Respondents had to affirm their consent before participation. Respondents who chose to participate in the survey, or chose to provide a Twitter ID may be different from those who do not participate or do not provide a Twitter ID. A comparison between the survey sample used in this paper and population-level values is provided in Supplementary Information A2.
Ethics oversight	New York University (NYU IRB 12-9058). Survey respondents consented to give YouGov their Twitter ID when participating in the survey, constituting the informed consent.

Note that full information on the approval of the study protocol must also be provided in the manuscript.

Field-specific reporting

Please select the one below that is the best fit for your research. If you are not sure, read the appropriate sections before making your selection.

☐ Life sciences ☒ Behavioural & social sciences ☐ Ecological, evolutionary & environmental sciences

For a reference copy of the document with all sections, see nature.com/documents/nr-reporting-summary-flat.pdf

Behavioural & social sciences study design

All studies must disclose on these points even when the disclosure is negative.

Study description	This study uses multi-wave panel data linked to respondents' Twitter accounts to examine the amount and concentration of US respondents' exposure to Russian foreign influence campaign accounts during the 2016 US presidential election; the characteristics of users who were exposed to Russian foreign influence accounts; and the relationship between exposure to Russian foreign influence accounts and changes (across survey waves) in political attitudes, polarization, and voting behavior.
Research sample	Respondents were drawn from YouGov's proprietary panel to be approximately representative of U.S. adult population. From this representative sample, respondents were asked for their Twitter IDs and consent for use in research. The sample was chosen to get as close a possible to a representative understanding of US citizens' political behavior on Twitter and its relationship to respondent characteristics.
Sampling strategy	Sampling strategy was to reflect a nationally representative U.S. sample. We collected responses from 3,500 respondents, of whom 1,496 provided their Twitter ID. Because the Russian foreign influence campaign was an unanticipated event, the characteristics of this large survey were not specifically calibrated to the research design used in this article.
Data collection	Data were collected online by the survey firm YouGov. Respondents could complete the survey privately, on their own time, on any appropriate web- and browser-enabled device.
Timing	Three waves of the survey took place between April and October 2016. Lists of respondents' friends were collected in April 2016. Tweets/retweets/quotes of respondents' friends were collected in December 2016.
Data exclusions	Analyses relied on 1496 respondents, who provided their Twitter identifiers. Of the 3500 respondents, 2004 were excluded for not providing their Twitter identifiers.
Non-participation	Recruitment of sample from YouGov's proprietary panel was handled by the vendor. We are unable to report a non-participation rate.
Randomization	Due to the nature of the object of study, the study uses observational panel data analysis, not randomization. The activity of the Russian foreign influence campaign during the 2016 US election was, at the time, unknown, and randomization of assignment to receive posts from an election interference campaign during a democratic election would both not be possible, and would be unethical.

Reporting for specific materials, systems and methods

We require information from authors about some types of materials, experimental systems and methods used in many studies. Here, indicate whether each material, system or method listed is relevant to your study. If you are not sure if a list item applies to your research, read the appropriate section before selecting a response.

Materials & experimental systems

n/a	Involved in the study
☒	☐ Antibodies
☒	☐ Eukaryotic cell lines
☒	☐ Palaeontology and archaeology
☒	☐ Animals and other organisms
☒	☐ Clinical data
☒	☐ Dual use research of concern

Methods

n/a	Involved in the study
☒	☐ ChIP-seq
☒	☐ Flow cytometry
☒	☐ MRI-based neuroimaging
